# Locally manufactured point-of-care CD19 chimeric antigen receptor T-cell therapy for paediatric relapsed/refractory B-cell acute lymphoblastic leukaemia: a single-centre feasibility experience

**DOI:** 10.3389/fonc.2026.1900851

**Published:** 2026-10-27

**Authors:** Wilson Yau Ki Chan, Alex Wing Kwan Leung, Hui Zhang, Daniel Ka Leung Cheuk, Pamela Pui Wah Lee, Gina Yi Jen Jiang, Chung Kai Wong, Maggie Zi Ying Chow, Yvonne Chu, Margaret Heung Ling Ng, Linda Hanssens, Liang Gao, Kam Tong Leung, Chi Kong Li

**Affiliations:** 1 Department of Paediatrics and Adolescent Medicine, Hong Kong Children’s Hospital, Hong Kong, Hong Kong SAR, China; 2 Department of Paediatrics, The Chinese University of Hong Kong, Hong Kong, Hong Kong SAR, China; 3 Department of Paediatrics and Adolescent Medicine, The University of Hong Kong, Hong Kong, Hong Kong SAR, China; 4 Institute of Biotechnology, The Chinese University of Hong Kong, China, Hong Kong, Hong Kong SAR, China; 5 The Hong Kong Institute of Biotechnology Limited, Hong Kong, Hong Kong SAR, China; 6 Department of Anatomical & Cellular Pathology, The Chinese University of Hong Kong, Hong Kong, Hong Kong SAR, China; 7 Miltenyi Biomedicine GmbH, Bergisch Gladbach, Germany

**Keywords:** CD19, immunotherapy, adoptive therapy, leukaemia, B-cell, point-of-care systems, receptors, chimeric antigen

## Abstract

**Background:**

The high cost of commercial CD19-targeted chimeric antigen receptor T-cell (CAR-T) therapy for relapsed/refractory B-cell acute lymphoblastic leukaemia (B-ALL) is prohibitive for many centres and families worldwide. It is imperative to develop a cost-effective, high-quality local manufacturing system to make CAR-T a financially feasible point-of-care (POC) production option for patients.

**Objective and study design:**

This was a single-centre, open-label, non-randomised phase II clinical trial aimed at evaluating the efficacy and safety of the locally manufactured autologous anti-CD19 CAR-T cells, generated using the CliniMACS Prodigy system, in the treatment of relapsed/refractory B-ALL. The production efficiency, efficacy, and toxicity of CAR-T therapy were evaluated.

**Results:**

A total of 6 patients were recruited, including 3 male and 3 female patients. Five patients were recruited upon first relapse, while one was recruited upon second relapse. Two patients had central nervous system involvement upon relapse, as determined by flow cytometry (CNS2). The median age at CAR-T infusion was 9 years (range 4–14 years). The median time from the most recent relapse to CAR-T infusion was 5.33 months (range 4.58–5.83 months). The median percentage of blasts was 5% (range 3%–93%) in bone marrow before CAR-T infusion. All patients received intravenous cyclophosphamide 1,000 mg/m^2^ and fludarabine 120 mg/m^2^ for lymphodepletion. The median cell dose was 2.67 × 10^6^ CAR-positive viable T cells/kg (range 2.3–2.95 × 10^6^/kg). The median transduction efficiency was 42.86% (range 33.89%–62.1%) and the median viability of CD3+ cells was 96.22% (range 95.61%–96.78%). The median viral copy number was 2.03 (range 1.72–2.48). All products were intact with no visible damage or leakage. The median filled volume per bag was 28.96 mL (range 15.8–64.01), with a median number of viable CAR-T cells of 76 × 10^6^ cells (range 47 to 174 × 10^6^ cells) and a median concentration of viable CAR-T cells of 2.71 × 10^6^/mL (range 2.41–2.99 × 10^6^/mL). No aggregates or endotoxins (<0.500 EU/mL) were detected in any of the products. Tests for bacterial growth and mycoplasma by quantitative PCR (qPCR) were all negative. Five patients experienced grade 1 cytokine release syndrome (CRS), while one patient experienced grade 3 CRS. CRS occurred at a median of 1.5 days (range 0–6 days) after infusion and resolved by a median of 6.5 days (range 5–16 days) after infusion with the use of a median of 3 doses (range 1–8 doses) of tocilizumab. At a median follow-up of 9 months, all patients remained in remission. The median progression-free survival was not reached. All patients were administered intravenous immunoglobulin replacement every 4 weeks after CAR-T infusion.

**Conclusion:**

Locally manufactured POC CAR-T may be a feasible option for providing potentially safe, timely, and cost-effective therapy for B-ALL upon first relapse, based on preliminary Hong Kong data from a small number of patients. Further validation in larger studies is recommended to validate scalability, long-term persistence, and cost-effectiveness.

## Introduction

Despite recent advances in the treatment of acute lymphoblastic leukaemia (ALL), 10%–20% of patients fail frontline treatment and relapse, and some die from disease progression or treatment-related complications (1). CD19-targeted chimeric antigen receptor (CAR) T-cell therapy has emerged as a new modality to treat B-lineage haematological malignancies. In July 2017, tisagenlecleucel (CTL 019; Kymriah) became the first CAR-T approved by the United States Food and Drug Administration (FDA) for the treatment of relapsed/refractory (R/R) B-cell ALL (B-ALL) in children and young adults, based on an international multi-centre phase II study showing an 83% complete remission (CR) rate and an overall survival (OS) rate of 89% at 6 months in a heavily pretreated population that included patients with prior allogeneic haematopoietic stem cell transplantation (HSCT) (2).

While CAR-T therapy has yielded impressive results in the treatment of B-ALL, the complex manufacturing process and tight quality control have limited its scalability and global availability (3, 4). The FDA mandates good manufacturing practice (GMP)-certified facilities for manufacturing viral vectors and CAR-T to ensure quality and sterility. The vein-to-vein time between apheresis of lymphocytes from patients at a local treatment centre and infusion of manufactured, cryopreserved CAR-T delivered from overseas GMP-certified facilities typically takes 4 to 8 weeks, and some patients inevitably die of disease progression while awaiting CAR-T manufacturing and delivery. Furthermore, due to the high costs of manufacturing and delivery, Kymriah is priced at USD 475,000 at present, which is prohibitive for many centres and families. In addition to the challenges in manufacturing cost, capacity, logistics, and timeline challenges, substantial global disparities in CAR-T access, even in high-income and upper-middle-income countries and across social classes, have been identified due to various barriers, including cost disparities, regional availability, racial/ethnic inequalities, and socio-economic disparities (5–7).

It is imperative to develop a cost-effective, high-quality local manufacturing system to make CAR-T therapy a financially feasible point-of-care (POC) production option for patients (3). The fully automated Prodigy^®^ system developed by Miltenyi Biotec has demonstrated safety, feasibility, high efficacy, and low toxicity in the production and infusion of fresh non-cryopreserved autologous CAR-T cells (8). Recently, a POC study conducted in India, the VELCART trial, demonstrated the initial success of the Prodigy system, which has low production costs (9). This manuscript is an early report of a phase II clinical trial involving 6 paediatric subjects in Hong Kong using the same manufacturing system and vector as a phase I clinical trial published by a Russian group (10), with a target cell dose of 0.5–3.0 × 10^6^ CAR-positive viable T cells/kg (range 2.3-2.95 x 10e6/kg) (see **Table 1**).

**Table 1:** Basic demographics, disease status before CAR-T infusion, CAR-T infusion details, and patient outcomes after CAR-T infusion.

Patient	1	2	3	4	5	6
**Subject study number**	CUCART-001	CUCART-005	CUCART-006	CUCART-013	CUCART-015	CUCART-017
Sex	Male	Female	Male	Female	Male	Female
Age at initial diagnosis (years)	12.19	0.10	5.86	8.33	2.99	4.10
Initial diagnosis	Ph-like B-cell ALL	Infant B-cell ALL	B-cell ALL	B-cell ALL	B-cell ALL	B-cell ALL
Risk stratification	Intermediate risk	Intermediate risk	Low risk	Intermediate risk	Intermediate risk	Low risk
Cytogenetics	51,XY,+5,+6,+8, inv(9)(p12q13), t(9;14)(p24;q13), +21,+22[20]/ 46,XY,inv(9)(p12q13)[6]	46,XX,t(4;11;11) (q21;q23;p11.2)[7]/ 46,XX[13]	57,XY,+X,dup(1)(q21q41),t(2;22)(p11.2;q11.2),+4,+5,+6,+10,+14,add(16)(q24),+17,+18,+21,+21, +mar[9]/46,XY[11]	46,XX,add(9)(p13), add(9)(p22), add(12)(p13) [12]/ 45,XX,t(1;9)(p13;q13),9,add(12)(p13)[4]/ 46,XX[5]	53,XY,+X,+4,+6,+10, +14,+21,+21[2]/ 53,idem,dup(1)(q12q32),add(6)(q25)[14]/ 53,idem,dup(1) (q32q21)[6]/53,idem,dup(1)(q32q21),add(4)(q21)[6]/46,XY[2]	45,X,-X[22]/ 46,XX[2]
Molecular genetics	C14orf93-JAK2, CTX, in frame, pathogenic	KMT2A::AFF1 fusion	Not detected	MEF2D rearrangement	Increased copy number of MEF2D and RUNX1 (1q duplication and tetrasomy 21)	ETV6-RUNX1 fusion
CNS leukaemia at initial diagnosis	No	No	No	No	No	No
Upfront treatment protocol	CCCG ALL 2020	Interfant 06	CCCG ALL 2015	CCCG ALL 2020	CCCG ALL 2020	CCCG ALL 2020
Indication for CAR T-cell infusion	Early BM relapse of Ph-like B-cell ALL during maintenance	Relapsed Infant B-cell ALL with KMT2A rearrangement	Second relapsed B-cell ALL (BM+testis)	Very early BM relapse	Very early relapse of BM and CNS	Isolated BM relapse 17 months after Tx completion
Age at diagnosis of relapse (years)	14.52	5.25	1^st^ BM relapse: 9.38; 2^nd^ BM + testicular relapse: 13.72	9.88	4.58	8.11
CNS leukaemia at relapse	No	Yes, CNS2 (13% by Flow cytometry)	No	No	Yes, CNS 2 (10% by Flow cytometry)	No
Relapse treatment protocol	CCCG relapsed ALL 2017 induction	HKPHOSG relapsed ALL 2007 pre-phase and F1 block	1^st^ relapse: CCCG relapsed ALL 2017 2^nd^ relapse: HKPHOSG relapsed ALL 2007 pre-phase and modified F1 block	HKPHOSG relapsed ALL 2007	HKPHOSG relapsed ALL 2007, then Homoharringtonin + venetolax	HKPHOSG relapsed ALL 2007
Bridging chemotherapy prior to apheresis and lymphodepletion	Nil	ALL IC-BFM 2002 protocol III phase 2 CAM	Protocol III modified CAM	Modified protocol 1b (reduced dose) CYM (500mg/m2 D1 Ara-C (75mg/m2/d D3-6	Homoharringtonin + venetolax	Modified protocol Ib, given 2 Ara-C blocks
Prior HSCT before CAR-T infusion	No	No	No	No	No	No
Prior use of blinatumomab or inotuzumab before CAR-T infusion	No	No	No	No	No	No
Disease status prior to CAR-T	BM: 9% blasts MRD by flow: 7.67%	BM: 7% blasts MRD by PCR 0.81%	BM: 3% blasts MRD by flow: 3.8%	BM < 5% blasts MRD by PCR 1.69%	BM: 93% blasts MRD by flow: 88%	BM: 3% blasts MRD by flow: 0.12%
Lansky Performance Scale prior to CAR T-cell infusion	100	100	100	100	100	100
Co-morbidities or organ dysfunction prior to CAR T-cell infusion	Nil	Nil	Nil	Nil	Nil	Nil
Age at CAR T-cell infusion (years)	14.7	5.4	13.9	10.0	4.7	8.3
Treatment BW (kg)	50	16	60	35	20	20
Time from latest relapse to CAR T-cell therapy (months)	5.67	5.58	5.08	4.58	4.67	5.83
Vein-to-vein time (days)	14	14	14	14	14	14
Transduction efficiency (% of CAR+ cells in viable CD3+ cells)	43.31	33.89	62.10	42.4	51.35	40.69
Cell dose (x10^6^ CAR-positive viable T-cells/kg)	2.3	2.95	2.9	2.64	2.6	2.7
Remission status post CAR T-cells infusion	CR2, MRD negative	CR2, MRD negative	CR3, MRD negative	CR2, MRD negative	CR2, MRD negative	CR2, MRD negative
Day of discharge post CAR T-cells infusion	10	87	13	15	34	14
Complications post CAR-T cells infusion, related management and outcomes						
CRS (max grade)	Yes (G1)	Yes (G1)	Yes (G1)	Yes (G1)	Yes (G3)	Yes (G1)
ICANS (max grade)	No (G0)	No (G0)	No (G0)	No (G0)	Yes (G2)	No (G0)
IEC-HS	No	No	No	No	Yes	No
Day of onset of CRS post-CAR T-cell infusion	4	1	1	2	0	6
Day of CRS resolution post-CAR T-cell infusion	6	6	7	5	16	8
Usage of tocilizumab	Yes, 1 dose (400mg/dose i.e. 8mg/kg)	Yes, 3 doses (190mg/dose i.e. 12mg/kg)	Yes, 2 doses (480mg/dose i.e. 8mg/kg)	Yes, 4 doses (280mg/dose i.e. 8mg/kg)	Yes, 8 doses (280mg/dose i.e. 12mg/kg)	Yes, 3 doses (240mg/dose i.e. 12mg/kg)
Steroid use	No	No	No	No	Yes, dexa 2mg/kg/day in 4 div doses from day +5 to +10, then 1mg/kg/day in 4 div doses till day +12 then 0.5mg/kg/day in 2 div doses till day +15	No
ICU admission	No	No	No	No	Yes, admitted for total 10 days from day +7 to +17 for HFNC max 25L/min	No
Other intervention(s)	No	No	No	No	Anakinra Emapalumab Ruxolitinib	No
Disease remission status and CAR-T cell persistence and function post-infusion						
**Bone marrow** Day +30 Day +90 Day +180 9 months 12 months	Morphological and molecular CR Morphological CR Molecular MRD + * Morphological CR Molecular MRD + * Morphological and molecular CR Morphological and molecular CR	Morphological and molecular CR Morphological and molecular CR Morphological and molecular CR NA Morphological and molecular CR	Morphological and molecular CR Morphological and molecular CR Morphological and molecular CR NA Morphological and molecular CR	Morphological and molecular CR Morphological and molecular CR T/F T/F T/F	Morphological and molecular CR Morphological and molecular CR T/F T/F T/F	Morphological and molecular CR T/F T/F T/F T/F
**CSF negative** Day +30 Day +90 Day +180 9 months 12 months	Yes NA NA NA Yes	Yes Yes Yes NA Yes	Yes Yes Yes NA Yes	Yes Yes T/F T/F T/F	Yes Yes T/F T/F T/F	Yes T/F T/F T/F T/F
**PB B-cell aplasia** Day +30 Day +90 Day +180 12 months 18 months	Yes Yes Yes Yes Yes	Yes Yes Yes Yes T/F	Yes Yes Yes Yes T/F	Yes Yes T/F T/F T/F	Yes Yes T/F T/F T/F	Yes T/F T/F T/F T/F
**CAR T-cells in PB** Day +7 Day +14 Day +30 Day +60 Day +90 Day +180 9 months 12 months 18 months	27.5% 29.3% 20.25% NA 0.65% 0.19% NA 0.052% <0.11%	NA NA 44.7% NA 6.1% 0.43% <0.11% 0.69% T/F	68.9% NA 79% NA 3.8% 0.56% 0.443% 0.112% T/F	18.38% 10.39% 2.23% NA 0.079% T/F T/F T/F T/F	NA 61% 34.86% T/F T/F T/F T/F T/F T/F	40.5% NA 38% T/F T/F T/F T/F T/F T/F
**CAR T-cells in CSF** Day +7 Day +14 Day +30 Day +60 Day +90 Day +180 9 months 12 months 18 months	NA NA <0.11% NA <0.13% <0.22% NA ND* T/F	NA NA >33% NA 21% <0.42% NA <0.18% T/F	NA NA <0.11% NA <0.13% <0.22% NA ND* T/F	NA NA 17% NA <3.85% T/F T/F T/F T/F	NA NA 4.6% T/F T/F T/F T/F T/F T/F	NA NA 73% T/F T/F T/F T/F T/F T/F
Need of HSCT as consolidation post CAR-T infusion	No	No	No	No	No	No
Duration of follow-up (months)	22.9	16.8	14.0	6.7	4.0	2.8

ALL: acute lymphoblastic leukaemia; BM: bone marrow; BW: body weight; CAR: chimeric antigen receptor; CCCG: Chinese Children’s Cancer Group; CNS: central nervous system; CR: complete remission; CR2: complete remission (second); CR3: complete remission (third); CRS: cytokine release syndrome; CSF: cerebrospinal fluid; dexa: dexamethasone; div: divided; G: grade; HFNC: high flow nasal cannula; HKPHOSG: Hong Kong Paediatric Haematology Oncology Study Group; HLH: haemophagocytic lymphohistiocytosis; ICANS: immune effector-cell associated neurotoxicity syndrome; ICU: intensive care unit; IEC-HS: immune effector cell hyperinflammatory syndrome; kg: kilograms; L/min: litre(s) per minute; max: maximum; MRD: minimal residual disease; NA: not available; ND: not detected; PB: peripheral blood; Sx: symptom* Limit of detection 0.65%.

## Patients and methods

### Study design

This was a single-centre, open-label, non-randomised phase II clinical trial aimed at evaluating the efficacy and safety of the locally manufactured autologous anti-CD19 CAR-T therapy generated by the CliniMACS Prodigy system in treating R/R B-ALL. The production efficiency (i.e., the successful production of CAR-T cells in ≥90% of manufacturing procedures), the efficacy, and the toxicity of the CAR-T therapy were evaluated. Autologous CAR-T cells were manufactured locally by the Hong Kong Institute of Biotechnology (HKIB), a certified local GMP facility, while the phase II clinical trial to confirm the efficacy and safety of the locally manufactured CAR-T product was conducted at the Hong Kong Children’s Hospital (HKCH). Analyses of CAR-T, cytokine profiles, and vector copies were performed by the Department of Anatomical and Cellular Pathology at the Prince of Wales Hospital.

### Inclusion criteria

Paediatric patients (aged below 19 years old) with relapsed or refractory CD19-positive B-ALL, either in first or subsequent relapse, or relapse after allogeneic HSCT (≥3 months after HSCT and off immunosuppressants for ≥1 month), or persistent minimal residual disease (MRD)-positive disease were included. Patients with active leukaemia who had developed significant organ impairment (except for active clinically significant central nervous system (CNS) dysfunction) and could not tolerate conventional chemotherapy were also eligible. Eligible subjects were required to have a Lansky performance scale of >50 (for those ≤16 years old) or an Eastern Cooperative Oncology Group performance score of ≤2 (for those >16 years old). Before study enrolment, all patients and their family members were fully informed about the availability of the market-approved CAR-T product (Kymriah) as a potential alternative treatment option for eligible patients.

### Manufacture of CAR-T

The apheresis product was transported to the HKIB advanced therapy product (ATP) GMP Centre under ambient conditions at 15–28°C with a target yield of 2 × 10^9^ CD3^+^ cells and a minimum yield of 0.6 × 10^9^. The target leukocyte collection was 2 × 10^8^ mononuclear cells/kg. Cells were transferred to the CliniMACS Prodigy^®^ system for T-cell enrichment using CD4^+^ and CD8^+^ magnetic beads, followed by activation using CD3 and CD28 agonists (T-cell TransAct™). Activated T cells were subsequently transduced with a self-inactivating lentiviral vector encoding a CAR targeting CD19. The vector construct for the CD19-specific CAR-T cell production was developed and validated in preclinical studies by Lentigen, a Miltenyi Biotec company. A novel CAR was constructed by combining the CD19-targeting single-chain fragment variable (scFv) sequence FMC63 in-frame with a CD8 hinge sequence, a transmembrane region sequence from TNF receptor superfamily member 19 (TNFRSF19), a co-stimulatory domain derived from 4-1BB/CD137, and a CD3ζ activation domain. All sequences, except for the FMC63 scFv, which was derived from heavy and light chains of a murine antibody, were of human origin. The cells were then cultured, expanded, and tested for transduction efficiency, sterility, and immunophenotype before preparation for patient use as previously described (11). Manufacturing was undertaken in a GMP-qualified environment in compliance with the Pharmaceutical Inspection Co-Operation Scheme (PIC/S) Guide to Good Manufacturing Practice.

The CliniMACS^®^ Prodigy system utilised apheresed mononuclear cells as the starting material and provided a fully automated, integrated platform for CAR-T manufacturing, including T-cell selection, activation, expansion, lentiviral transduction, and cell harvest. CAR-T production was performed in a Grade C clean room environment using the CliniMACS^®^ Prodigy closed manufacturing system, in accordance with PIC/S GMP guidelines. The system employed a closed, single-use disposable tubing set to minimise contamination risk. Release testing for CAR-T included Gram stain, sterility, mycoplasma, endotoxin level, replication-competent lentivirus, vector copy number, cell viability, percentage of CD3^+^ cells, percentage of CAR^+^ cells, viable CAR-T cell concentration, and residual B cells. After product release by the Authorized person (AP) registered under the Pharmacist and Poisons Regulations, who is listed by the Pharmacy and Poisons Board and certifies compliance for advanced therapy products, the CAR-T cells were shipped fresh to the recipients by a validated transportation process under refrigerated conditions at 2–8°C. Under standard manufacturing conditions, the CAR-T cells were administered 14 days after initiation of apheresis.

### Quality assurance

Product release was governed by predefined acceptance criteria encompassing identity (CAR expression by flow cytometry), viability (≥70%), total and viable cell dose, sterility, endotoxin levels (<5 IU/kg), and the absence of adventitious agents. Sterility testing was performed using both rapid microbiological methods and conventional culture-based assays; interim results from in-process samples could be used for initial product certification to enable the timely infusion of fresh CAR-T cells, with final certification based on culture results obtained after infusion. Potency was assessed using a standardised *in vitro* cytotoxicity assay against CD19-expressing target cells, with acceptance defined as ≥30% specific lysis at a 5:1 effector-to-target ratio.

Vector safety was monitored by quantifying vector copy number using quantitative PCR (qPCR), with an upper release limit of five copies per genome. Testing for replication-competent lentivirus (RCL) was performed using a sensitive cell-based assay with PCR-based confirmation, and no RCL was detected in any batch. Throughout the manufacturing process, robust chain of identity and chain of custody procedures were implemented, including dual verification of patient identifiers, barcode-labelled containers, and electronic tracking from collection to infusion.

Out-of-specification (OOS) products were managed according to a predefined risk-assessment framework; OOS batches were reviewed by the quality unit and the clinical team and, where clinically justified, could be released with the appropriate documentation and regulatory notification. Manufacturing deviations and batch failures were systematically recorded, investigated through root cause analysis, and addressed with corrective and preventive actions. Continuous process monitoring and trend analysis were employed to ensure manufacturing consistency and to minimise the incidence of deviations and batch failures.

### Bridging chemotherapy

Patients were recruited soon after diagnosis of relapsed ALL. They were administered standard reinduction chemotherapy (12). If patients had a good response to the first phase of reinduction, the intensity of the second phase of chemotherapy would be reduced to avoid excessive toxicities. Mild bridging chemotherapy with intermediate-dose cyclophosphamide (500 mg/m^2^) and cytarabine (75 mg/m^2^/day) for 4 days per week was administered until lymphocyte collection (13). For more resistant cases, chemotherapy was tailored according to drug profiling (14). The target was to achieve a bone marrow blast percentage of <20% before CAR-T therapy.

### Lymphodepletion conditioning and fresh CAR-T infusion

Once the test results for in-process CAR-T were satisfactory on day 5 of manufacturing, lymphodepleting chemotherapy was administered, comprising intravenous (IV) fludarabine 30 mg/m^2^ daily for 4 days (days -6 to -3) and IV cyclophosphamide 500 mg/m^2^ daily for 2 days, starting with the first dose of fludarabine. After 2 rest days post-chemotherapy, fresh non-cryopreserved CAR-T cells were infused intravenously over 5–30 minutes through a central venous catheter. The target cell dose was 0.5–3.0 × 10^6^ CAR-positive viable T cells per kilogram of body weight. Premedication with paracetamol and antihistamines was administered 30 minutes before the infusion.

### Clinical monitoring and management after CAR-T infusion

All patients were hospitalised for the administration of the CAR-T products and a subsequent observation period. Their clinical status and progress (including physical examination, vital signs, and neurological assessment), along with peripheral blood results (e.g., complete blood count, biochemistry, coagulation profile, immunoglobulin levels, lymphocyte subsets, CAR-T persistence assessment, serum cytokine profile, and acute phase inflammatory markers, including C-reactive protein and ferritin) were monitored before, during, and after CAR-T infusion. Adverse events and serious adverse events were recorded and managed according to standard guidelines. Cytokine release syndrome (CRS) was graded and managed according to the American Society for Transplantation and Cellular Therapy (ASTCT) consensus grading for CRS and Neurologic Toxicity Associated with Immune Effector Cells (15). Patients were observed as inpatients for at least 7 days after treatment. Neutropenic fever and tumour lysis syndrome were managed according to institutional guidelines. Prophylaxis for herpes virus, fungal infections, and Pneumocystis jirovecii pneumonia was administered according to institutional guidelines. Irradiated red blood cells or platelet concentrates were administered whenever patients required blood product support until 3 months after CAR-T infusion or until immune recovery, whichever occurred later. Serum immunoglobulin levels were monitored monthly, and IV immunoglobulin replacement at 500 mg/kg would be administered monthly from day 30 onwards or as indicated.

### Post-CAR-T therapy disease evaluation

Examination of bone marrow and cerebrospinal fluid was performed on day 28 and at 3, 6, and 12 months after CAR-T infusion. Lymphocyte subsets on day 28 and then monthly for 6 months were checked for B-cell aplasia. The persistence of CD19 CAR-T cells in the bone marrow and peripheral blood was assessed using immunohistochemistry, flow cytometry, and qPCR at 1, 3, 6, 9, and 12 months after CAR-T infusion, and at relapse if indicated. MRD was also monitored. Patients were regularly followed up for at least 2 years after CAR-T therapy for any late complications.

### Statistical analyses

This study was primarily descriptive in nature with respect to patient-level outcomes due to the small sample size. Continuous variables were presented as median (range), and categorical data were expressed as frequencies (percentages).

### Research ethics

This study was conducted in compliance with the International Council for Harmonisation - Good Clinical Practice (ICH-GCP) guidelines and the Declaration of Helsinki. Ethical approval (HKCH-REC-2020-063) and informed consent from the study subjects were obtained. The clinical trial was registered at ClinicalTrials.gov NCT06462248.

### Funding and insurance

This research study was funded by the Hong Kong Special Administrative Region Sponsor Innovation and Technology Fund. Patient insurance was purchased through the Joint CUHK-NTEC Clinical Research Management Office.

## Study results

### Basic demographics

A total of 6 patients were recruited, including 3 male and 3 female patients. Five patients were recruited at the first relapse of CD19-positive B-ALL, while one was recruited at the second relapse. Two out of five relapses had central nervous system involvement with CNS2 status (blasts identified by flow cytometry only). The median age at CAR-T infusion was 9 years (range 4–14 years). The median time from the most recent relapse to CAR-T infusion was 5.33 months (range 4.58–5.83 months). The median percentage of blasts was 3% (range 3%–93%) in bone marrow before CAR-T infusion. The vein-to-vein time between autologous apheresis and CAR-T infusion was uniformly 14 days for all patients. The median cell dose infused was 2.64 × 10^6^ CAR-positive viable T cells/kg (range 2.3–2.95 × 10^6^/kg).

### Manufacturing metrics

Production efficiency was 100%, with a 100% manufacturing success rate. The median transduction efficiency was 42.86% (range 33.89%–62.1%) and the median viability of CD3+ cells was 96.22% (range 95.61%–96.78%). The median viral copy number was 2.03 (range 1.72–2.48). All products were intact with no visible damage or leakage. The median filled volume per bag was 28.96 mL (range 15.8–64.01), with a median number of viable CAR-T cells of 76 × 10^6^ cells (range 47 to 174 × 10^6^ cells) and a median concentration of viable CAR-T cells of 2.71 × 10^6^/mL (range 2.41–2.99 × 10^6^/mL). No aggregates or endotoxins (<0.500 EU/mL) were detected in any of the products. Tests for bacterial growth and mycoplasma by qPCR were all negative (see **Table 2**).

**Table 2:** Certificate of analysis (COA) report for the CUCART product.

Batch No.	0124002	0125002	0125003	0125010	0126001	0126003
Study No.	CUCART-001	CUCART-005	CUCART-006	CUCART-013	CUCART-015	CUCART-017
Initial	PTC	NCY	PYK	HKH	LHS	WWY
Apheresis Product on Day 0						
Total cell viability (%)	96.8	99.06	99.47	98.83	98.16	99.42
Total cell concentration (x 10^7^ cells/mL)	6.31	3.18	3.77	3.42	1.63	1.72
Viable CD3^+^ cells (x 10^9^ cells)	6.75	2.48	4.07	2.34	1.82	1.7
Viable TNC (x 10^9^ cells)	12.5	3.87	7.20	3.95	2.46	2.62
Ratio of CD3^+^/TNC (%)	54.0	64.13	56.53	59.17	73.7	64.91
Volume of Apheresis Product (mL)	212	123	200	125	162	161
Approx volume of Citrate / ACD-A (mL)	26	13	22	14	18	17
Volume of Apheresis Product net (mL)	186	110	178	111	144	144
Isolated T-Cells						
Viability of CD3^+^ cells (%)	99.38	99.62	99.50	99.29	99.34	99.12
Viable CD3^+^ cells (x 10^8^ cells)	9.75	9.98	12.5	14.6	12.0	9.5
%CD3^+^	95.76	95.16	96.45	95.9	96.9	94.63
%CD4^+^	44.52	39.11	53.34	33.74	47.55	36.31
%CD8^+^	47.47	53.68	40.83	51.50	47.8	55.77
CUCART19 on Day 5						
Viability of CD3^+^ cells (%)	97.41	93.97	95.16	93.38	89.67	93.78
%CAR^+^ in viable CD3^+^ cells	45.21	38.75	59.55	47.29	49.69	40.11
%CD3^+^	99.59	99.67	99.50	99.15	99.67	99.71
%CD4^+^	50.86	28.96	52.58	31.87	48.98	29.41
%CD8^+^	47.18	68.98	45.11	62.69	47.61	68.55
The Day 5 sample sent out for sterility test	Yes	Yes	Yes	Yes	Yes	Yes
COA Product Report on Day 13						
Appearance	Absence of aggregates	Absence of aggregates	Absence of aggregates	Absence of aggregates	Absence of aggregates	Absence of aggregates
Product bag integrity	Intact with no visible damage or leakage	Intact with no visible damage or leakage	Intact with no visible damage or leakage	Intact with no visible damage or leakage	Intact with no visible damage or leakage	Intact with no visible damage or leakage
Filled volume per bag (mL)	48.89	15.8	64.01	37.03	20.89	19.44
Vector copy number (copies)	2.12	1.72	2.48	1.95	2.08	1.98
Cell viability (%)	96.01	96.38	95.58	95.43	95.65	96.35
Viability of CD3+ cells (%)	96.36	96.59	96.08	95.61	95.91	96.78
% of CAR+ cells in viable CD3+ cells	43.31	33.89	62.10	42.4	51.35	40.69
Concentration of viable CAR-T cells (x10^6^/mL)	2.41	2.99	2.72	2.58	2.7	2.8
Number of viable CAR-T cells (x10^6^ cells)	118	47	174	96	56	55
Mycoplasma by qPCR	Negative	Negative	Negative	Negative	Negative	Negative
Endotoxin (EU/mL)	<0.500	<0.500	<0.500	<0.500	<0.500	<0.500
Sterility of CART19 -D5	No Growth	No Growth	No Growth	No Growth	No Growth	No Growth

### Clinical outcomes

#### Disease remission

All patients achieved complete remission with negative MRD 1 month after CAR-T. All patients remained in continuous remission after CAR-T therapy at a median follow-up of 9 months (range 2–22 months), with CAR-T persistence and B-cell aplasia (see **Table 1**; **Figure 1**). The median progression-free survival was not reached. The qPCR and flow cytometry data showed robust early expansion (Day 7–14 peaks of up to 120,000 copies/µg DNA), CAR-T persistence up to 12 months in several patients, and sustained B-cell aplasia in the majority of cases. The CD4/CD8 ratios were consistent with 4-1BB CAR-T biology (see **Supplementary Table S1**). All patients were administered IV immunoglobulin replacement every 4 weeks after CAR-T infusion (see **Supplementary Table S2**). No patients in the cohort had clinically significant or laboratory-proven bacterial, viral, fungal, or mycobacterial infections.

**Figure 1:**
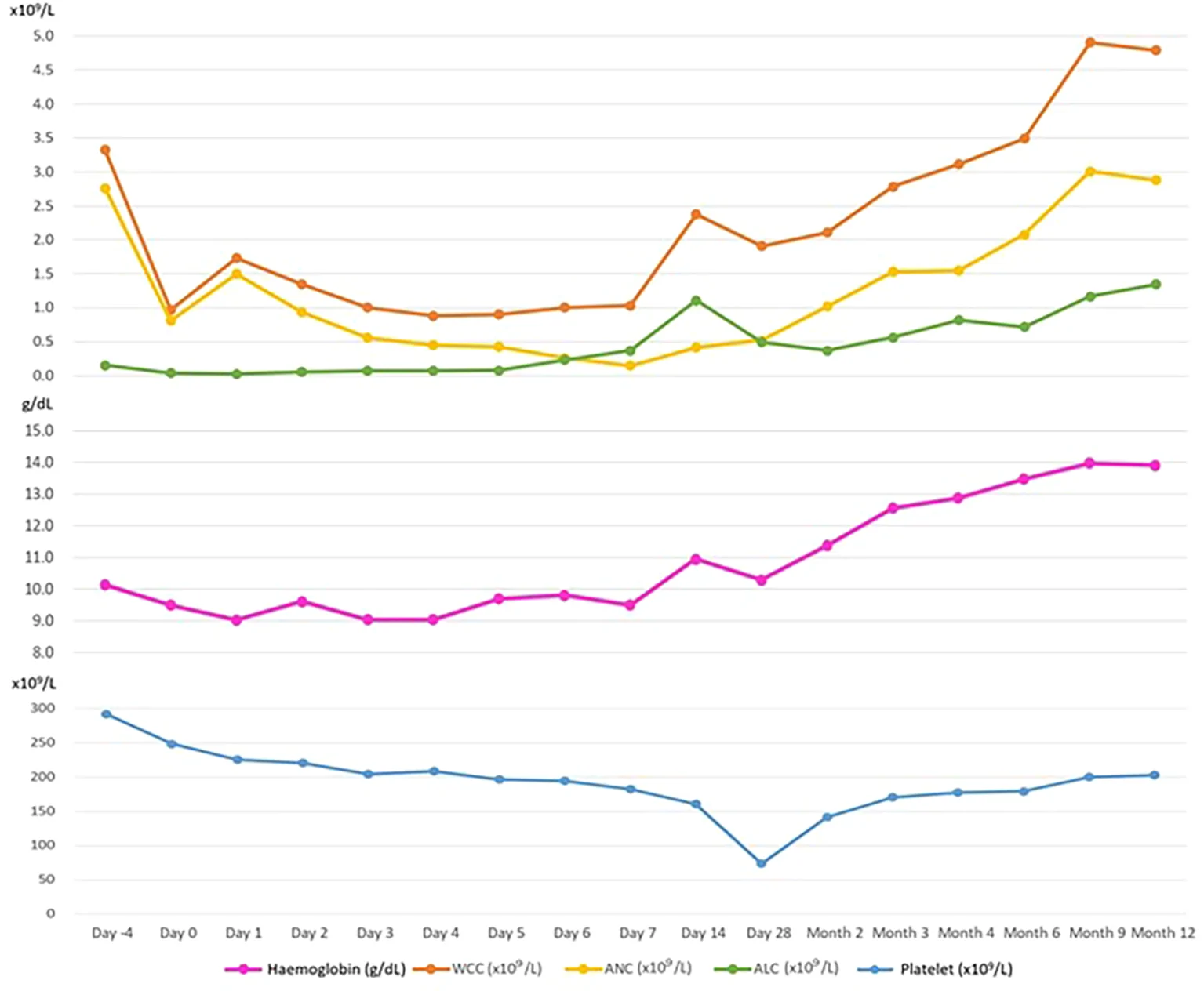
Serial trend of complete blood counts (mean values) after CAR-T infusion.

#### Cytokine release syndrome and immune effector cell-associated neurotoxicity syndrome

Five patients (subject study numbers CUCART-001, 005, 006, 013, and 017) experienced grade 1 CRS, while one patient (subject study number CUCART-015) experienced grade 3 CRS. CRS occurred at a median of 1.5 days (range 0–6 days) after infusion and resolved by a median of 6.5 days (range 5–16 days) after infusion with the use of a median of three doses (range 1–8 doses) of tocilizumab. The patient (subject study number CUCART-015) who developed grade 3 CRS also developed grade 4 immune effector cell-associated haemophagocytic lymphohistiocytosis-like syndrome (IEC-HS), presumably related to a high disease load (93% bone marrow blasts and 88% minimal residual disease by flow cytometry) (see **Table 1**). The patient presented with a high fever of up to 40°C on day +5, pulmonary oedema (with tachypnoea, desaturation, and oxygen dependency), hepatosplenomegaly (maximum liver span 15.3 cm at the mid-clavicular line, maximum spleen size 9.5 cm as documented sonographically on day +10), irritability with fluctuating sensorium (purposeless speech and screaming), hyperferritinaemia (maximum 599,720 pmol/L on day +10, reference range 9–151 pmol/L), hypertriglyceridaemia (maximum 9.5 mmol/L on day +11, reference range 0.5–2.5 mmol/L), hypofibrinogenaemia (lowest <0.5 g/L on day +9, reference range 1.7–3.5 g/L), elevated soluble CD25 (maximum 37,711 pg/mL on day +8, reference range 126–734 pg/mL), a significantly activated interferon gamma-mediated signalling pathway as reflected by elevated CXCL9 (maximum 1,553.1 pg/mL on day +8, reference range <77.7 pg/mL), elevated lactate dehydrogenase (maximum 13,398 IU/L on day +9, reference range ≤305 IU/L), transaminitis and cholestasis [maximum serum alanine aminotransferase 139 IU/L on day +9, reference range <39 IU/L, 3.6 times the upper limit of normal (ULN); serum aspartate aminotransferase 2,240 IU/L on day +9, reference range 29–53 IU/L, 42 × ULN, serum gamma glutamyltransferase 939 IU/L on day +11, reference range 3–22 IU/L, 43 × ULN; serum bilirubin 54 μmol/L on day +9, reference range ≤17 μmol/L, 3.2 × ULN], hyperammonaemia (maximum 148 μmol/L, reference range 21–50 μmol/L), coagulopathy [prolonged prothrombin time with a maximum of 20.1 seconds on day +9 (reference range 11.2–13.4 seconds); prolonged activated partial thromboplastin time of 38.9 seconds on day +9 (reference range 24–30 seconds), maximum internal normalised ratio 1.98 on day +9]. He required admission to the intensive care unit (ICU) and administration of dexamethasone, anakinra, emapalumab, and ruxolitinib (see **Supplementary Table S3**). All patients recovered from CRS and other complications and were discharged at a median of 14.5 days after CAR-T infusion (range 10–87 days) (see **Table 1**; **Figure 2**; **Supplementary Table S3**).

**Figure 2:**
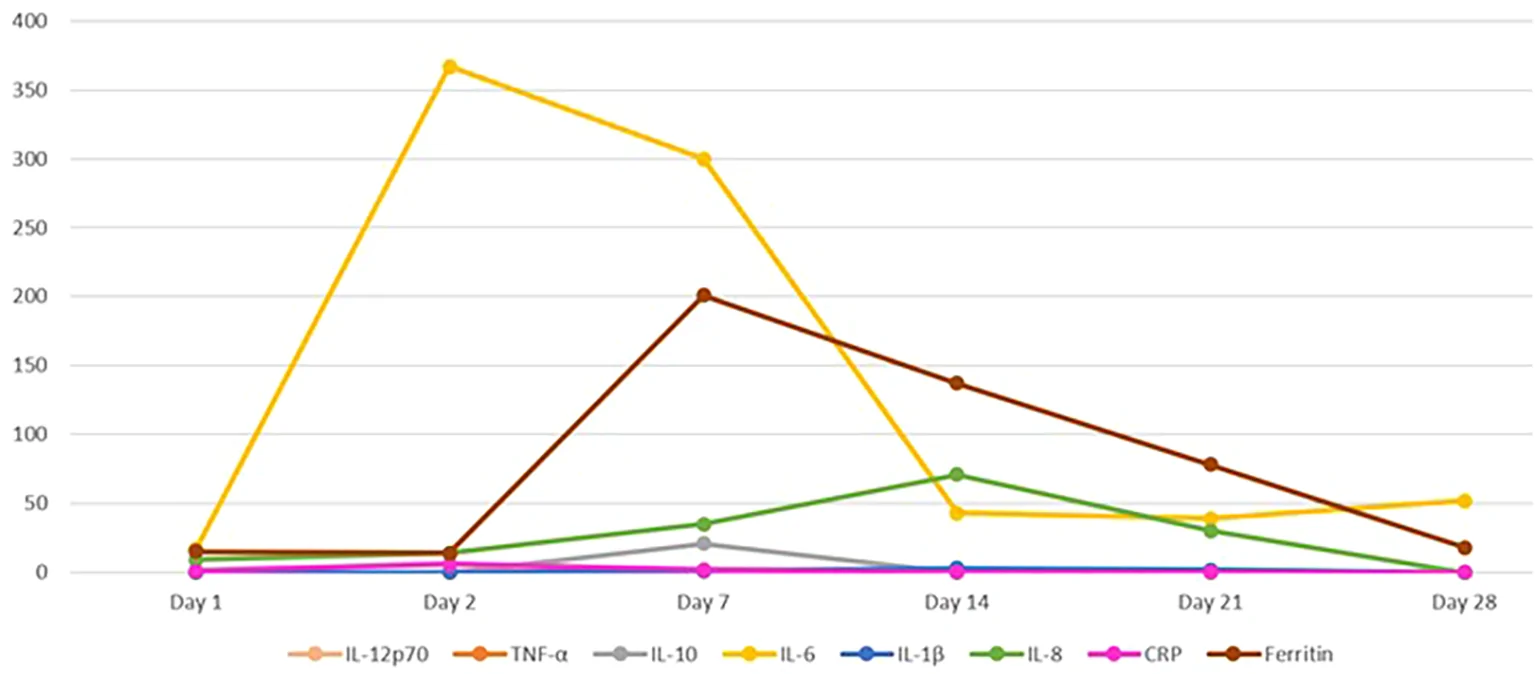
Serial trend of cytokine profile after CAR-T infusion.

#### Immune effector cell-associated haematotoxicity

In the local cohort, biphasic cytopenia, grade 4 neutropenia in all patients around day +7, gradual recovery by 2–3 months, no anaemia, and a platelet nadir/recovery pattern were noted. No patients developed transfusion-dependent anaemia, and only grade 1 thrombocytopenia was present on day 28 after CAR-T infusion and resolved spontaneously. Patients experienced a nadir of grade 4 neutropenia (absolute neutrophil count [ANC] <0.5 × 10^9^/L) on day 7 after CAR-T infusion, which persisted until day 28 after CAR-T infusion, followed by grade 3 neutropenia (ANC 0.5–1.0 × 10^9^/L) from 1 to 2 months after CAR-T infusion, and then grade 2 neutropenia (ANC 1.0–1.5 × 10^9^/L) from 2 to 3 months after CAR-T infusion. One patient had haematotoxicity with neutropenia that required prolonged granulocyte colony-stimulating factor (G-CSF) support until 3 months after CAR-T infusion and then resolved (see **Figure 3**).

**Figure 3:**
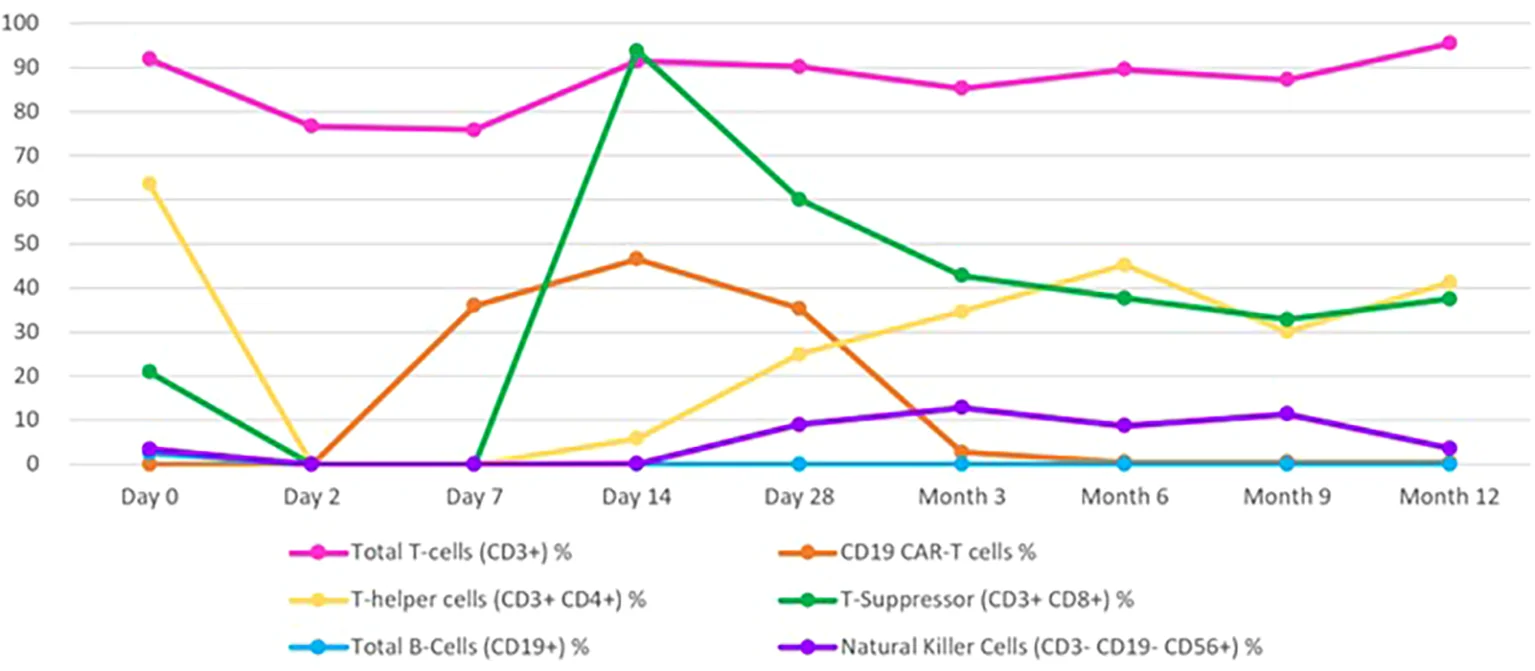
Serial lymphocyte immunophenotyping demonstrating CAR-T persistence and B-cell aplasia.

## Discussion

CAR-T therapy targeting CD19 has transformed treatment for R/R B-ALL, but commercial products may face delays from centralised manufacturing and cryopreservation. Locally manufactured POC CAR-T therapy may offer shorter timelines using fresh non-cryopreserved cells, and potentially enable earlier intervention in first-relapse settings traditionally reserved for allogeneic HSCT. POC CAR-T manufacturing using CliniMACS Prodigy has already been reported from India in the VELCART trial (9) and is not a new concept. The present manuscript adds to the published literature by focusing on a specific cohort of paediatric B-ALL in Hong Kong, with encouraging early results: all six patients achieved remission, with a good manufacturing success rate. The unique contributions include the paediatric setting, the local Hong Kong GMP platform, use in the setting of the first relapse, the presence of flow cytometric CNS disease, and fresh infusion logistics. This manuscript presented early feasibility experience with local CAR-T manufacturing in Hong Kong.

### Shorter vein-to-vein time with fresh cells

Commercial CAR-T usually requires 30–45 days from apheresis to infusion due to shipping, manufacturing, and quality control of cryopreserved products, with the risk of disease progression. Local POC production potentially shortens this to 2 weeks by using fresh, non-cryopreserved cells, presumably sparing shipping time and cryopreservation. As cryopreservation may impair cell viability and function, fresh cells may show better expansion and persistence (16–18).

### Application in first bone marrow relapse B-ALL and extramedullary relapse

Commercial CAR-T is licensed for second or later relapses. Patients with high-risk or intermediate-risk first relapse of CD19-positive B-ALL are now treated with intensive chemotherapy and blinatumomab and then proceed to allo-HSCT, which is associated with 20%–30% transplant-related mortality and complications such as graft-versus-host disease (19). Patients with low-risk CD19-positive B-ALL are treated with chemotherapy and blinatumomab, with maintenance treatment for up to 2 years (20). HSCT is not adopted routinely; the 2-year disease-free survival is only 54.4% (21). Local CAR-T can be used upon first relapse as a standalone treatment to achieve long-term remission, sparing high-risk transplant in responsive cases (22). Patients with suboptimal response, persistence of MRD, or early loss of B-cell aplasia after CAR-T therapy may be considered for allo-HSCT as a second-line treatment. Studies confirm high CR rates (>80%) in early relapse or MRD-positive B-ALL with CAR-T (23). For patients with extramedullary relapse involving the CNS or testes, local radiotherapy combined with chemotherapy and allo-HSCT might achieve a satisfactory survival outcome, but these patients would have long-term complications, including infertility, cognitive impairment, and second cancers. CAR-T therapy has been demonstrated to effectively control CNS and testicular relapse (24) and thus spare patients from later complications.

### Improved disease control via rapid process

With the availability of local CAR-T therapy, the timing of apheresis and CAR-T reinfusion may be better managed. Chemotherapy for the control of relapses may be offered as a less intensive regimen and may avoid early induction morbidity and mortality. Short apheresis-to-infusion times with local manufacturing allow low-dose chemotherapy bridges with minimal toxicity to maintain targeted blast levels, unlike the intensive regimens needed during prolonged commercial waits. This preserves disease control in aggressive B-ALL. Based on previous in-house experience and audit, 20%–30% of patients deteriorated before commercial infusion when only commercial CD19 CAR-T (Kymriah) was available. Rapid platforms enable CR rates of 88%–100% with limited toxicity.

### Variability in transduction efficiency and viable CAR-T cell numbers

Despite the impressive results of 100% production efficiency and a 100% manufacturing success rate for local manufacturing, it was noted that the transduction efficiency (33.89%–62.1%) and viable CAR-T cell numbers (47–174 × 10^6^ cells) had a large range of variability. As manufacturing was performed using an automated closed system with procedures that fully complied with the operational manual, the factors contributing to this variability remain uncertain and warrant further investigation in future studies.

### Selection of upfront and bridging chemotherapy

Patients diagnosed with relapsed B-ALL at our centre were treated uniformly with the prevailing protocol (Hong Kong Paediatric Haematology and Oncology Study Group (HKPHOSG) relapsed ALL 2007 or Chinese Children’s Cancer Group (CCCG) relapsed ALL 2017 protocol). Details of the chemotherapy regimen are provided in **Table 1**, **Figure 4**. The ALL IC-BFM 2002 (Acute Lymphoblastic Leukemia Intercontinental Berlin-Frankfurt-Münster 2002) protocol III phase 2 Cyclophosphamide, Ara-C (cytarabine), 6-Mercaptopurine/6-Thioguanine (also known as modified protocol 1b), comprising intravenous cyclophosphamide and cytarabine together with intrathecal methotrexate, cytarabine, and hydrocortisone, was the bridging chemotherapy of choice. Modifications were based on individual organ function, therapeutic efficacy, and toxicity profile. For those with suboptimal organ function, intolerance to bridging chemotherapy, or suboptimal disease control with first-line chemotherapy, drug profiling was employed to guide the choice of a bridging chemotherapeutic agent, such as the use of homoharringtonin and venetoclax in subject CUCART-015 (see **Table 1**). Omission of bridging chemotherapy may also be considered based on the clinical context and the physician’s decision, taking into account disease control status and logistical considerations (e.g., the availability of an apheresis machine, the stem cell laboratory, and manpower of apheresis nurses, and other factors), as in subject CUCART-001 (see **Table 1**).

**Figure 4:**
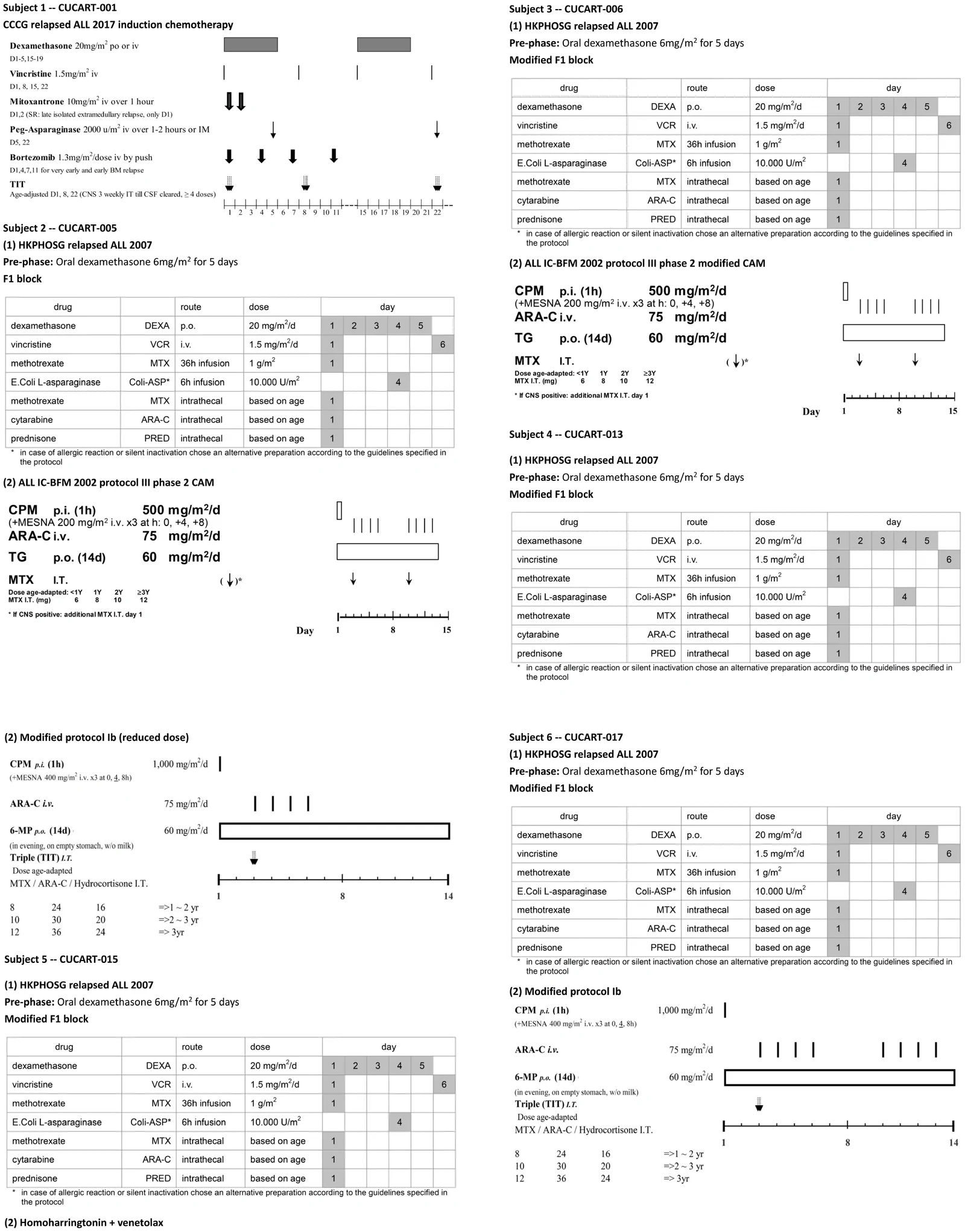
Upfront chemotherapy for relapsed B-ALL and bridging chemotherapy before apheresis and CAR-T cell infusion.

### Complications associated with CAR-T infusion

#### Cytokine release syndrome

CRS has emerged as the most severe toxicity of CAR-T therapy in previous clinical studies (2). CRS is a class-specific toxicity caused by a systemic inflammatory response to *in vivo* T-cell activation and expansion. It can be mild and self-limiting, with fever and flu-like symptoms such as myalgia, but can also become life-threatening, with hypotension, vascular leak, cytopenias, coagulopathy, and renal and respiratory failure. Severe CRS with cardiovascular dysfunction requiring vasoactive agents and/or respiratory failure requiring invasive mechanical ventilation was reported in 28% of paediatric and young adult patients treated with 4-1BB CAR-T in the largest clinical trial published to date (2). The pathogenesis of CRS was found to be driven by high serum release of interleukin-6 (IL-6), and management with the IL-6 receptor antagonist tocilizumab was reported to result in rapid clinical improvement.

#### Immune effector cell-associated neurotoxicity syndrome

Immune effector cell-associated neurotoxicity syndrome (ICANS) is another major complication after CAR-T treatment. Neurological toxicities such as headaches, confusion, hallucinations, seizures, aphasia, and behavioural abnormalities can occur both within and independent of CRS, generally within the first 2 weeks after CAR-T infusion. Approximately 77% of patients may develop CRS at a median of 3 days (range 1–22 days), and approximately half require ICU care with a median stay of 7 days (range 1–34 days) (2). Approximately 40% of patients may develop ICANS, which may present as seizures, delirium, and confusion. Grade 3 or higher CNS toxicities were observed in 25%–30% to >40% of patients in previous clinical studies. Neurological symptoms reported with anti-CD19 CAR-T therapy in the context of CRS typically occurred later during CRS and were not prevented by tocilizumab administered for severe CRS. In most patients, CNS toxicity was self-limiting within a median duration of 7 days; however, rare cases of severe and irreversible neurotoxicity have also been reported (2).

#### Immune effector cell-associated haemophagocytic lymphohistiocytosis-like syndrome

IEC-HS is a distinct hyperinflammatory toxicity after immune effector cell therapy according to ASTCT guidelines (25), separate from CRS but often clinically overlapping with it. It is characterised by unregulated, sustained immune activation that typically emerges as CRS resolves. Clinical presentations include prolonged or worsening fever, cytopenia, and hepatosplenomegaly. Biochemical features include hyperferritinaemia, hypertriglyceridaemia, hypofibrinogenaemia, coagulopathy, transaminitis, and end-organ damage, such as acute kidney injury and neurotoxicity. Early recognition and prompt treatment are critical, as delayed management of severe IEC-HS can lead to multiorgan failure and death. As prospective trial data are scarce, suggested management is largely based on consensus and expert opinion (25–28) and includes systemic steroids, ruxolitinib, anakinra, emapalumab, and other immunomodulatory approaches along with intensive supportive care. The patient (subject study number CUCART-015) who developed grade 3 CRS with grade 4 IEC-HS had a very high leukaemia load before CAR-T therapy, which might account for his serious clinical course and requirement for admission to the intensive care unit. The administration of dexamethasone, anakinra, emapalumab, and ruxolitinib resulted in a good response with complete recovery (27). The other four patients with a much lower disease burden only experienced grade 1 CRS, which resolved by a median of 6 days after infusion with the use of a median of three doses of tocilizumab. All patients survived without long-term sequelae.

#### Immune effector cell-associated haematotoxicity

Haematological toxicity is the most common adverse event after CAR-T therapy, with a cumulative 1-year incidence of 58% (Common Terminology Criteria for Adverse Events [CTCAE] grade ≥3) in real-world settings (29). It is characterised by a biphasic temporal course and is often prolonged (30–33). Risk factors include severe CRS/ICANS, cytopenia before initiation of lymphodepleting chemotherapy, and prior allogeneic stem cell transplantation (30, 32). Cytopenia predisposes patients to severe infectious complications and non-relapse mortality, which are the most frequent cause of non-relapse mortality (34).

The use of growth factors to manage early cytopenias after CAR-T cell therapy remains controversial. Current recommendations discourage the use of granulocyte-macrophage colony-stimulating factor (GM-CSF) due to its association in the pathogenesis in the pathogenesis of CRS and neuroinflammation (35). Initiation of G-CSF is recommended only after resolution CRS and/or ICANS (36). However, in a report by Galli et al., prophylactic G-CSF was used safely in 42 patients with grade 4 neutropenia on day 5 after CAR-T cell infusion, with no increased risk of CRS or ICANS and no negative impact on disease outcomes (37). For the local cohort, we administer G-CSF based on the clinical context, depending on whether the patient has neutropenic fever or not, instead of administering prophylactically based on neutrophil count alone. Autologous or allogeneic stem cell rescue may be considered where possible (31, 38). Other options include anti-inflammatory therapy (e.g., dexamethasone, IL-6- blocking therapy) and thrombopoietin receptor agonists (e.g., eltrombopag).

Autologous or allogeneic stem cell rescue may be considered where possible (31, 38). Other options include anti-inflammatory therapy (e.g., dexamethasone, IL-6-blocking therapy) and thrombopoietin receptor agonists (e.g., eltrombopag).

Infections are significant complication of CAR-T therapy as a result of prolonged neutropenia, long-term CD4 T-cell lymphopenia, or B-cell aplasia (39). The majority of infections occur early within the first 28 days, with bacterial infections being the most common, followed by viral and fungal infections. Late infections, especially with respiratory viruses, are also seen up to 90 days post-CAR-T therapy (39). The use of antiviral, antifungal, and Pneumocystis pneumonia (PCP) prophylaxis to reduce the risk of infections after CAR-T cell therapy is recommended; however, there is no consensus on the optimal choice and duration of prophylaxis. In the absence of high-risk factors, yeast-active prophylaxis may be sufficient, with pre-emptive monitoring for moulds recommended (40). For our institution, we employed micafungin as anti-fungal prophylaxis from infusion till neutrophil count >0.5 x 10_9_/L for 3 consecutive days without growth factor support, acyclovir from infusion till 6 months post-infusion with CD4 count >0.2 x 10_9_/L, co-trimoxazole as PCP prophylaxis from 1 week prior to infusion for at least 1 year post-infusion with CD4 count >0.2 x 10_9_/L. Monthly IV immunoglobulins would be given regularly post-infusion and as indicated when there is persistent severe hypogammaglobulinaemia and a history of recurrent infections. Re-vaccination would be considered at least 6-12 months post-CAR-T therapy, with decisions guided by post-vaccine antibody titres whenever available (39).

### Local success demonstrates efficacy and safety despite study limitations

This study highlights global healthcare equity in CAR-T therapy accessibility and strengthens the evidence base for local POC production of cell therapies. The proof-of-concept data are compelling, and the manufacturing approach is reproducible. Key limitations of this study include a small sample size of only six patients, a single-centre design, a short follow-up period, the absence of a comparator group, and selection bias. Despite the above limitations, a proof-of-concept local GMP-compliant CAR-T platform in Hong Kong successfully treated 6 paediatric patients with R/R B-ALL, achieving 100% remission, CAR-T persistence, and controllable CRS (predominantly grade 1–2). All cases remain in remission and have achieved molecular CR. This confirms that local production yields effective and safe CAR-T therapy, enhancing access in resource-limited settings. The literature supports CRS control and a correlation between persistence and durable remissions (>6–12 months) (41–43). This reported cohort provides further evidence supporting this claim. However, due to the small sample size, it is difficult to conclude whether the CD4/CD8 ratio was consistent with 4-1BB CAR biology and predicted durability as compared to the CD28/CD3ζ (28-z) design. Extended follow-up (e.g., a minimum of 2 years after CAR-T infusion), publication of longitudinal follow-up data, and multicentre validation trials are recommended.

## Conclusion

Based on preliminary Hong Kong data, local POC CAR-T therapy may be a feasible option offering timely, cost-effective therapy for first-relapse B-ALL with acceptable logistics and outcomes. Future trials should validate its scalability, long-term persistence, and cost savings. Integration into protocols could reduce reliance on HSCT, minimising mortality risks and late complications.

## Data Availability

The original contributions presented in the study are included in the article/**Supplementary Material**. Further inquiries can be directed to the corresponding authors.
