# Supplementary material for: Locally manufactured point-of-care CD19 chimeric antigen receptor T-cell therapy for paediatric relapsed/refractory B-cell acute lymphoblastic leukaemia: a single-centre feasibility experience
Source: Front Oncol. 2026 Oct 27;16:1900851. doi: 10.3389/fonc.2026.1900851 (PMC13749038; doi:10.3389/fonc.2026.1900851)
Supplement: Supplementary file 1 [file Table1.docx]

**Supplementary Table 1**	Serial lymphocyte immunophenotyping post CAR T-cell infusion

| **Study subject number** | **Time Point** | **Sample** | **Mean gag vector copies/μg genomic DNA** | **Total T-cells (CD3^+^)** | | **T-helper cells (CD3^+^ CD4^+^)** | | **T-Suppressor (CD3^+^ CD8^+^)** | | **Total B-Cells (CD19^+^)** | | **Natural Killer Cells (CD3^-^ CD19^-^ CD56^+^)** | | **CD4/CD8**  **Ratio** | **White Cell Count** | **Lymphocytes** | | **CD19 CAR-T cells** | |
| --- | --- | --- | --- | --- | --- | --- | --- | --- | --- | --- | --- | --- | --- | --- | --- | --- | --- | --- | --- |
|  |  |  |  | **%** | **x10^9^/L** | **%** | **x10^9^/L** | **%** | **x10^9^/L** | **%** | **x10^9^/L** | **%** | **x10^9^/L** |  | **x10^9^/L** | **%** | **x10^9^/L** | **%** | **x10^9^/L** |
| CUCART-001 | Day 0 | PB qPCR | 0 | 97.4 | 0.03 | 82.2 | 0.03 | 13.3 | 0.00 | 0.4 | 0.00 | 0.9 | 0.00 | 6.183 | 2.2 | 2 | 0.03 | UD | UD |
|  | Day 2 | PB qPCR | 0 | 96.90 | 0.08 | NA | NA | NA | NA | NA | NA | NA | NA | NA | 3.5 | 2 | 0.08 | 0.0 | 0.00 |
|  | Day 7 | PB qPCR | 31006.47 | 93.2 | 0.50 | NA | NA | NA | NA | NA | NA | NA | NA | NA | 2.0 | 27 | 0.54 | 22.8 | 0.1229 |
|  | Day 13 | PB qPCR | 30533.85 | 81.3 | 0.21 | NA | NA | NA | NA | NA | NA | NA | NA | NA | 1.2 | 22 | 0.26 | 33.4 | 0.0869 |
|  | Day 29 | PB qPCR | 23693.20 | 82.7 | 0.13 | 39.6 | 0.06 | 26.1 | 0.04 | 0.0 | 0.00 | 15.6 | 0.03 | 1.520 | 1.5 | 11 | 0.16 | 20.0 | 0.0324 |
|  | Mth 3 | PB qPCR | 244.09 | 76.5 | 0.23 | 45.1 | 0.13 | 19.8 | 0.06 | 0.0 | 0.00 | 20.2 | 0.06 | 2.281 | 2.0 | 15 | 0.30 | 0.5 | 0.0012 |
|  | Day 111 | PB qPCR | 147.15 | 81.8 | 0.41 | 45.8 | 0.23 | 25.6 | 0.13 | 0.0 | 0.00 | 16.2 | 0.08 | 1.792 | 1.2 | 43 | 0.50 | 0.164 | <0.01 |
|  | Mth 6 | PB qPCR | 100 | 91.0 | 0.75 | 48.6 | 0.40 | 34.0 | 0.28 | 0.0 | 0.00 | 7.4 | 0.06 | 1.429 | 1.9 | 43 | 0.82 | * | NA |
|  | Mth 9 | PB qPCR | 1047.22 | 91.5 | 1.06 | 47.3 | 0.55 | 33.2 | 0.39 | 0.0 | 0.00 | 7.5 | 0.09 | 1.424 | 3.2 | 36 | 1.16 | 0.961 | 0.0102 |
|  | Mth 12 | PB qPCR | 0 | 93.7 | 1.01 | 42.4 | 0.46 | 32.2 | 0.35 | 0.0 | 0.00 | 5.7 | 0.06 | 1.318 | 4.3 | 25 | 1.08 | 0.052 | 0.0006 |
| CUCART-005 | Day 0 | PB qPCR | 0 | 93.8 | 0.03 | 55.8 | 0.02 | 25.3 | 0.01 | 2.4 | 0.00 | 1.6 | 0.00 | 2.206 | 0.9 | 4 | 0.04 | UD | NA |
|  | Day 2 | PB qPCR | 0 | 94.9 | 0.05 | NA | NA | NA | NA | NA | NA | NA | NA | NA | 0.8 | 6 | 0.05 | UD | NA |
|  | Day 7 | PB qPCR | 57157.17 | 89.0 | 0.37 | NA | NA | NA | NA | NA | NA | NA | NA | NA | 0.8 | 55 | 0.42 | 65.2 | NA |
|  | Day 14 | PB qPCR | 93125.61 | 92.6 | 2.90 | NA | NA | NA | NA | NA | NA | NA | NA | NA | 5.8 | 54 | 3.13 | 92.399 | NA |
|  | Day 28 | PB qPCR | 51779.81 | 94.2 | 0.51 | 28.2 | 0.15 | 62.9 | 0.34 | 0.0 | 0.00 | 5.0 | 0.03 | 0.449 | 3.0 | 18 | 0.54 | 44.7 | NA |
|  | Day 28 | BM | BM | 84.0 | 0.74 | NA | NA | NA | NA | NA | NA | NA | NA | NA | 4.1 | 22 | 0.88 | 45.8 | NA |
|  | Mth 3 | PB qPCR | 1728.51 | 90.8 | 0.35 | 29.1 | 0.12 | 56.3 | 0.22 | 0.0 | 0.00 | 7.3 | 0.03 | 0.518 | 2.8 | 14 | 0.40 | 6.537 | NA |
|  | Mth 6 | PB qPCR | 201.97 | 96.9 | 0.59 | 48.1 | 0.29 | 44.6 | 0.27 | 0.0 | 0.00 | 1.8 | 0.01 | 1.077 | 2.0 | 30 | 0.61 | 0.452 | NA |
|  | Mth 9 | PB qPCR | NA | NA | NA | <0.11 | NA | NA | NA | NA | NA | NA | NA | NA |  |  | 1.50 | <0.11 | NA |
|  | Mth 12 | PB qPCR | 561.81 | 97.3 | 1.51 | 40.1 | 0.62 | 42.8 | 0.66 | 0.0 | 0.00 | 1.5 | 0.02 | 0.936 | 5.5 | 28 | 1.55 | 0.692 | 0.0101 |

| CUCART-006 | Day 0 | PB qPCR | 0 | 95.4 | 0.02 | 79.5 | 0.02 | 13.4 | 0.00 | 1.7 | 0.00 | 1.5 | 0.00 | 5.928 | 0.9 | 2 | 0.02 | UD | UD |
| --- | --- | --- | --- | --- | --- | --- | --- | --- | --- | --- | --- | --- | --- | --- | --- | --- | --- | --- | --- |
|  | Day 2 | PB qPCR | 0 | 98.6 | 0.04 | NA | NA | NA | NA | NA | NA | NA | NA | NA | 1.3 | 3 | 0.04 | UD | UD |
|  | Day 7 | PB qPCR | 49269.18 | 97.9 | 0.77 | NA | NA | NA | NA | NA | NA | NA | NA | NA | 2.1 | 38 | 0.79 | 68.899 | 0.5331 |
|  | Day 16 | PB qPCR | 120840.06 | 96.9 | 1.85 | NA | NA | NA | NA | NA | NA | NA | NA | NA | 3.0 | 63 | 1.92 | 94.08 | 1.7450 |
|  | Day 28 | PB qPCR | 104381.13 | 92.7 | 1.00 | 14.1 | 0.15 | 77.6 | 0.84 | 0.0 | 0.00 | 6.9 | 0.07 | 0.182 | 2.8 | 38 | 1.08 | 74.973 | 0.7646 |
|  | Mth 3 | PB qPCR | 344.16 | 83.3 | 0.38 | 33.6 | 0.15 | 40.9 | 0.19 | 0.0 | 0.00 | 15.1 | 0.07 | 0.820 | 4.1 | 11 | 0.46 | 3.889 | 0.0147 |
|  | Mth 6 | PB qPCR | 230.65 | 80.8 | 0.47 | 38.8 | 0.22 | 34.5 | 0.20 | 0.0 | 0.00 | 17.1 | 0.10 | 1.123 | 6.6 | 9 | 0.58 | 0.514 | 0.0023 |
|  | Mth 9 | PB qPCR | 349.61 | 82.9 | 0.62 | 43.0 | 0.32 | 32.4 | 0.24 | 0.0 | 0.00 | 15.3 | 0.12 | 1.327 | 5.9 | 13 | 0.75 | 0.443 | 0.0028 |
|  | Mth 12 | T/F | T/F | 84.5 | 0.80 | 44.2 | 0.42 | 32.4 | 0.31 | 0.0 | 0.00 | 13.5 | 0.13 | 1.365 | 6.8 | 14 | 0.95 | 0.112 | 0.0009 |
| CUCART-013 | Day 0 | PB qPCR | 0 | 89.2 | 0.03 | 42.7 | 0.01 | 42.9 | 0.01 | 1.7 | 0.00 | 7.1 | 0.00 | 0.996 | 1.4 | 2 | 0.03 | UD | UD |
|  | Day 5 | PB qPCR | 7637.29 | 88.1 | 0.12 | NA | NA | NA | NA | NA | NA | NA | NA | NA | 0.4 | 39 | 0.14 | 18.380 | 0.0221 |
|  | Day 15 | PB qPCR | 12885.52 | 86.6 | 0.60 | NA | NA | NA | NA | NA | NA | NA | NA | NA | 1.4 | 51 | 0.69 | 10.397 | 0.0624 |
|  | Day 31 | PB qPCR | 2951.45 | 83.8 | 0.6 | 24 | 0.17 | 55.9 | 0.40 | 0.0 | 0.00 | 15.1 | 0.11 | 0.429 | 1.9 | 37 | 0.72 | 2.228 | 0.0135 |
|  | Mth 3 | PB qPCR | 100 | 90.3 | 1.08 | 30.9 | 0.37 | 54.3 | 0.65 | 0.0 | 0.00 | 8.8 | 0.11 | 0.570 | 2.5 | 48 | 1.20 | 0.079 | 0.0008 |
|  | Mth 6 | T/F | T/F | T/F | T/F | T/F | T/F | T/F | T/F | T/F | T/F | T/F | T/F | T/F | T/F | T/F | T/F | T/F | T/F |
|  | Mth 9 | T/F | T/F | T/F | T/F | T/F | T/F | T/F | T/F | T/F | T/F | T/F | T/F | T/F | T/F | T/F | T/F | T/F | T/F |
|  | Mth 12 | T/F | T/F | T/F | T/F | T/F | T/F | T/F | T/F | T/F | T/F | T/F | T/F | T/F | T/F | T/F | T/F | T/F | T/F |
| CUCART-015 | Day 0 | PB qPCR | 0 | 90.8 | 0.02 | 56.5 | 0.01 | 15.7 | 0.00 | 0.4 | 0.00 | 7.9 | 0.00 | 3.603 | 0.2 | 15 | 0.03 | UD | UD |
|  | Day 2 | PB qPCR | 0 | BD | BD | BD | BD | BD | BD | BD | BD | BD | BD | BD | 0.1 | BD | BD | UD | UD |
|  | Day 6 | PB qPCR | 521.05 | BD | BD | BD | BD | BD | BD | BD | BD | BD | BD | BD | 0.1 | BR | BR | UD | UD |
|  | Day 10 | NA | NA | NA | NA | NA | NA | NA | NA | NA | NA | NA | NA | NA | 8.8 | ** | 0.13 | 74.933 | 6.53 |
|  | Day 14 | PB qPCR | 108362.63 | 99.7 | 0.26 | 5.9 | 0.02 | 93.8 | 0.25 | 0.0 | 0.00 | 0.1 | 0.00 | 0.063 | 0.3 | 80 | 0.26 | 2.630 | 0.0069 |
|  | Day 22 | NA | NA | 98.6 | 0.41 | NA | NA | NA | NA | NA | NA | NA | NA | NA | 0.5 | 78 | 0.41 | 70.038 | 0.2854 |
|  | Day 29 | PB qPCR | 64232.66 | 97.4 | 0.20 | 18.7 | 0.04 | 77.9 | 0.16 | 0.0 | 0.00 | 2.2 | 0.00 | 0.240 | 0.4 | 54 | 0.20 | 34.854 | 0.0664 |
|  | Mth 3 | T/F | T/F | T/F | T/F | T/F | T/F | T/F | T/F | T/F | T/F | T/F | T/F | T/F | T/F | T/F | T/F | T/F | T/F |
|  | Mth 6 | T/F | T/F | T/F | T/F | T/F | T/F | T/F | T/F | T/F | T/F | T/F | T/F | T/F | T/F | T/F | T/F | T/F | T/F |
|  | Mth 9 | T/F | T/F | T/F | T/F | T/F | T/F | T/F | T/F | T/F | T/F | T/F | T/F | T/F | T/F | T/F | T/F | T/F | T/F |
|  | Mth 12 | T/F | T/F | T/F | T/F | T/F | T/F | T/F | T/F | T/F | T/F | T/F | T/F | T/F | T/F | T/F | T/F | T/F | T/F |

| CUCART-017 | Day 0 | PB qPCR | 0 | 84.8 | 0.01 | 64.6 | 0.01 | 14.9 | 0.00 | 8.6 | 0.00 | 1.7 | 0.00 | 4.33 | 0.9 | 2 | 0.02 | UD | UD |
| --- | --- | --- | --- | --- | --- | --- | --- | --- | --- | --- | --- | --- | --- | --- | --- | --- | --- | --- | --- |
|  | Day 2 | PB qPCR | 0 | 93.1 | 0.04 | NA | NA | NA | NA | NA | NA | NA | NA | NA | 1.1 | 4 | 0.04 | UD | UD |
|  | Day 7 | PB qPCR | 34041.21 | 86.7 | 0.19 | NA | NA | NA | NA | NA | NA | NA | NA | NA | 0.6 | 36 | 0.22 | 40.625 | 0.0774 |
|  | Day 14 | PB qPCR | 117420.49 | 91.6 | 0.77 | NA | NA | NA | NA | NA | NA | NA | NA | NA | 1.8 | 47 | 0.84 | 81.816 | 0.6273 |
|  | Day 28 | PB qPCR | T/F | 83.8 | 0.22 | 22.3 | 0.06 | 44.2 | 0.11 | 0 | 0 | 15.4 | 0.04 | 0.505 | 0.7 | 36 | 0.26 | 38.339 | 0.08 |
|  | Mth 3 | T/F | T/F | T/F | T/F | T/F | T/F | T/F | T/F | T/F | T/F | T/F | T/F | T/F | T/F | T/F | T/F | T/F | T/F |
|  | Mth 6 | T/F | T/F | T/F | T/F | T/F | T/F | T/F | T/F | T/F | T/F | T/F | T/F | T/F | T/F | T/F | T/F | T/F | T/F |
|  | Mth 9 | T/F | T/F | T/F | T/F | T/F | T/F | T/F | T/F | T/F | T/F | T/F | T/F | T/F | T/F | T/F | T/F | T/F | T/F |
|  | Mth 12 | T/F | T/F | T/F | T/F | T/F | T/F | T/F | T/F | T/F | T/F | T/F | T/F | T/F | T/F | T/F | T/F | T/F | T/F |

BD: below detection limit; BM; bone marrow; BR: below reliable detection threshold; mth: month; NA: not applicable; PB: peripheral blood; qPCR; quantitative polymerase chain reaction; T/F: to follow; UD: undetected

* detected but not quantifiable, limit of detection (LOD) 0.05%

** predominantly atypical lymphocytes
