# Supplementary material for: Locally manufactured point-of-care CD19 chimeric antigen receptor T-cell therapy for paediatric relapsed/refractory B-cell acute lymphoblastic leukaemia: a single-centre feasibility experience
Source: Front Oncol. 2026 Oct 27;16:1900851. doi: 10.3389/fonc.2026.1900851 (PMC13749038; doi:10.3389/fonc.2026.1900851)
Supplement: Supplementary file 2 [file Table2.docx]

**Supplementary Table 2**	Serial cytokine profile post CAR T-cell infusion

| **Subject study number** | **Time point post CAR T-cell infusion** | **IL-12p70**  **(pg/mL)**  **[Ref <7.8]** | **TNF-α**  **(pg/mL)**  **[Ref <10.0]** | **IL-10**  **(pg/mL)**  **[Ref <7.8]** | **IL-6**  **(pg/mL)**  **[Ref <3.1]** | **IL-1β**  **(pg/mL)**  **[Ref <7.2]** | **IL-8**  **(pg/mL)**  **[Ref <10.0]** | **CXCL10/IP-10**  **(pg/ml)**  **[Ref 232-1019]** | **CCL2/MCP1**  **(pg/ml)**  **[Ref 18-152]** | **CXCL9/MIG**  **(pg/ml)**  **[Ref 37-463]** | **CCL5/RANTES**  **(pg/ml)**  **[Ref 10349-46704]** |
| --- | --- | --- | --- | --- | --- | --- | --- | --- | --- | --- | --- |
| CUCART-001 | Day 0 | <20 | <20 | <20 | <20 | <20 | <20 | NA | NA | NA | NA |
|  | Day 1 | <20 | <20 | <20 | <20 | <20 | <20 | NA | NA | NA | NA |
|  | Day 2 | <20 | <20 | <20 | <20 | <20 | <20 | NA | NA | NA | NA |
|  | Day 7 | <20 | <20 | <20 | 32.44 | <20 | <20 | NA | NA | NA | NA |
|  | Day 13 | <20 | <20 | <20 | 24.35 | <20 | <20 | NA | NA | NA | NA |
|  | Day 29 | <20 | <20 | <20 | 90.41 | <20 | <20 | NA | NA | NA | NA |
| CUCART-005 | Day 0 | <20 | <20 | <20 | <20 | <20 | <20 | NA | NA | NA | NA |
|  | Day 1 | <20 | <20 | <20 | <20 | <20 | <20 | NA | NA | NA | NA |
|  | Day 2 | <20 | <20 | <20 | <20 | <20 | <20 | NA | NA | NA | NA |
|  | Day 7 | <20 | <20 | <20 | 43.82 | <20 | 22.22 | NA | NA | NA | NA |
|  | Day 14 | <20 | <20 | <20 | 53.06 | <20 | <20 | NA | NA | NA | NA |
|  | Day 28 | <20 | <20 | <20 | 35.37 | <20 | <20 | NA | NA | NA | NA |
| CUCART-006 | Day 0 | <20 | <20 | <20 | <20 | <20 | <20 | NA | NA | NA | NA |
|  | Day 1 | <20 | <20 | <20 | 50.5 | <20 | 25.98 | NA | NA | NA | NA |
|  | Day 2 | <20 | <20 | <20 | 352.51 | <20 | <20 | NA | NA | NA | NA |
|  | Day 7 | <20 | <20 | <20 | 36.41 | <20 | <20 | NA | NA | NA | NA |
|  | Day 16 | <20 | <20 | <20 | 22.79 | <20 | <20 | NA | NA | NA | NA |
|  | Day 28 | <20 | <20 | <20 | 30.61 | <20 | <20 | NA | NA | NA | NA |
| CUCART-013 | Day 5 | UD | UD | UD | 203.9 | 7.6 | 21.3 | NA | NA | NA | NA |

| CUCART-015 | Day 2 | UD | UD | UD | 1115.6 | UD | 51.6 | 539.6 | 1229.1 | 332.0 | 935.9 |
| --- | --- | --- | --- | --- | --- | --- | --- | --- | --- | --- | --- |
|  | Day 6 | UD | UD | 110.8 | 1428.7 | UD | 142.2 | 912.8 | 399.8 | 2027.9 | 457.49 |
|  | Day 10 | UD | UD | 11.4 | 86.8 | UD | 429.7 | OR | 278.1 | OR | 870.73 |
|  | Day 14 | UD | UD | UD | 73.6 | 11.6 | 282.6 | 55.7 | 79.1 | 618.1 | 159.0 |
|  | Day 22 | 1.0 | 0.1 | 1.4 | 39.0 | 1.8 | 30.2 | 38.6 | 35.8 | 84.4 | 489.5 |
| CUCART-017 | Day 7 | 7.2 | 14.1 | 17.4 | 53.5 | UD | 25.9 | 610 | 48.1 | 692 | 12371 |

ml: millilitre(s); NA: not available; OR: out of range (higher than the highest standard); pg: picogram(s); ref: reference range; UD: undetectable

Note: Lower limit of quantification (LLOQ) of the assay for all cytokines is 20 pg/mL.
