# Supplementary material for: Locally manufactured point-of-care CD19 chimeric antigen receptor T-cell therapy for paediatric relapsed/refractory B-cell acute lymphoblastic leukaemia: a single-centre feasibility experience
Source: Front Oncol. 2026 Oct 27;16:1900851. doi: 10.3389/fonc.2026.1900851 (PMC13749038; doi:10.3389/fonc.2026.1900851)
Supplement: Supplementary file 3 [file Table3.docx]

**Supplementary Table 3**	Serial haematology and biochemistry parameters post CAR T-cell infusion

| Subject study number | Time  point | Hb  (g/dl) | WCC  (x10^9^/L) | ANC  (x10^9^/L) | ALC  (x10^9^/L) | Platelet  (x10^9^/L) | CRP  (mg/L) | Ferritin  (pmol/L) | ALT (IU/L)  (x ULN) | Fibrinogen  (g/L) | Creatinine  (μmol/L) | Creatinine  (mg/dL) | LDH (IU/L) | EASIX score |
| --- | --- | --- | --- | --- | --- | --- | --- | --- | --- | --- | --- | --- | --- | --- |
| CUCART-  001 | Screening | 11.4 | 5.39 | 3.18 | 1.10 | 393 | 4.1 | NA | 1.49 | 4.03 | 40 | 0.45 | 117 | 0.13 |
|  | Pre cell collection | 12.7 | 5.10 | 3.04 | 1.08 | 335 | NA | NA | 2.22 | 3.49 | 46 | 0.52 | 152 | 0.24 |
|  | Day -4 | 11.6 | 4.95 | 4.24 | 0.09 | 200 | NA | NA | 1.33 | 3.27 | 39 | 0.44 | 130 | 0.29 |
|  | Day 0 | 11.7 | 1.73 | 1.38 | 0.08 | 220 | NA | NA | 1.96 | 2.57 | 38 | 0.43 | 138 | 0.27 |
|  | Day 1 | 11.8 | 3.69 | 3.03 | 0.04 | 235 | 1.1 | 1946 | 1.86 | 2.51 | 42 | 0.48 | 138 | 0.28 |
|  | Day 2 | 11.7 | 3.18 | 1.87 | 0.07 | 263 | 2.8 | 1611 | 1.63 | 2.62 | 40 | 0.45 | 138 | 0.24 |
|  | Day 3 | 11.7 | 3.18 | 1.77 | 0.14 | 281 | 2.1 | 1514 | 1.73 | 2.65 | 42 | 0.48 | 143 | 0.24 |
|  | Day 4 | 12.1 | 2.36 | 1.59 | 0.08 | 300 | 1.9 | 1546 | 1.76 | 2.52 | 47 | 0.53 | 141 | 0.25 |
|  | Day 5 | 12.1 | 2.40 | 1.39 | 0.06 | 257 | 2.7 | 1479 | 1.41 | 2.68 | 52 | 0.59 | 135 | 0.31 |
|  | Day 6 | 11.9 | 1.46 | 0.20 | 0.28 | 220 | 13 | 1454 | 1.04 | 3.34 | 46 | 0.52 | 146 | 0.35 |
|  | Day 7 | 12.0 | 1.78 | 0.22 | 0.42 | 286 | 6.3 | 1607 | 0.96 | 2.85 | 51 | 0.58 | 145 | 0.29 |
|  | Day 13 | 13.1 | 1.21 | 0.35 | 0.28 | 351 | <0.6 | 1400 | 0.67 | 1.83 | 44 | 0.50 | 149 | 0.21 |
|  | Day 29 | 13.0 | 1.63 | 0.91 | 0.16 | 121 | <0.6 | 1476 | 0.65 | 1.55 | 43 | 0.49 | 229 | 0.92 |
|  | Month 2 | 14.3 | 1.82 | 1.10 | 0.28 | 241 | NA | NA | 0.53 | 2.35 | 51 | 0.58 | 152 | 0.36 |
|  | Month 3 | 14.7 | 2.06 | 1.38 | 0.32 | 218 | NA | NA | 0.43 | 2.48 | 47 | 0.53 | 174 | 0.42 |
|  | Month 4 | 14.7 | 1.26 | 0.33 | 0.52 | 224 | NA | NA | 0.33 | NA | 45 | 0.51 | 154 | 0.35 |
|  | Month 6 | 14.8 | 1.87 | 0.45 | 0.91 | 235 | NA | NA | 0.24 | 2.4 | 42 | 0.48 | 131 | 0.26 |
|  | Month 9 | 14.9 | 3.13 | 1.58 | 1.10 | 254 | NA | NA | 0.22 | 2.58 | 48 | 0.54 | 152 | 0.32 |
|  | Month 12 | 15.0 | 4.25 | 2.56 | 1.21 | 270 | NA | NA | 0.22 | 3.77 | 49 | 0.55 | 303 | 0.62 |

| Subject study number | Time  point | Hb  (g/dl) | WCC  (x10^9^/L) | ANC  (x10^9^/L) | ALC  (x10^9^/L) | Platelet  (x10^9^/L) | CRP  (mg/L) | Ferritin  (pmol/L) | ALT (IU/L)  (x ULN) | Fibrinogen  (g/L) | Creatinine  (μmol/L) | Creatinine  (mg/dL) | LDH (IU/L) | EASIX score |
| --- | --- | --- | --- | --- | --- | --- | --- | --- | --- | --- | --- | --- | --- | --- |
| CUCART-  005 | Screening | 9.8 | 1.85 | 0.14 | 1.63 | 191 | NA | NA | 6.95 | ND | 19 | 0.21 | 177 | 0.20 |
|  | Pre cell collection | 9.7 | 2.57 | 0.94 | 1.02 | 228 | NA | NA | 0.51 | 3.84 | 15 | 0.17 | 175 | 0.13 |
|  | Day -4 | 9.7 | 1.64 | 1.35 | 0.09 | 370 | <0.6 | 391 | 0.33 | 2.63 | 10 | 0.11 | 190 | 0.06 |
|  | Day 0 | 8.8 | 0.98 | 0.75 | 0.05 | 344 | <0.6 | ND | 0.38 | 2.26 | 14 | 0.16 | 171 | 0.08 |
|  | Day 1 | 9.0 | 1.15 | 0.86 | 0.05 | 330 | <0.6 | 333 | 0.36 | 2.31 | 14 | 0.16 | 175 | 0.08 |
|  | Day 2 | 9.2 | 0.93 | 0.59 | 0.06 | 345 | 1.0 | 283 | 0.31 | 2.72 | 14 | 0.16 | 182 | 0.08 |
|  | Day 3 | 9.0 | 0.84 | 0.37 | 0.07 | 327 | 1.2 | 245 | 0.33 | 2.83 | 18 | 0.2 | 189 | 0.12 |
|  | Day 4 | 9.0 | 1.02 | 0.37 | 0.1 | 311 | 1.3 | 261 | 0.31 | 3.00 | 18 | 0.2 | 197 | 0.13 |
|  | Day 5 | 9.0 | 0.84 | 0.22 | 0.09 | 304 | 1.3 | ND | 0.31 | ND | 16 | 0.18 | 201 | 0.12 |
|  | Day 6 | 9.5 | 1.15 | 0.25 | 0.34 | 291 | 1.0 | 319 | 0.33 | 3.49 | 20 | 0.23 | 200 | 0.16 |
|  | Day 7 | 9.3 | 0.94 | 0.03 | 0.49 | 251 | 0.6 | 283 | 0.31 | 3.26 | 14 | 0.16 | 188 | 0.12 |
|  | Day 14 | 10.4 | 5.39 | 0.85 | 2.45 | 143 | <0.6 | 151 | 0.28 | 1.46 | 14 | 0.16 | 223 | 0.25 |
|  | Day 28 | 7.7 | 2.63 | 0.15 | 0.44 | 6 | <0.6 | 492 | 0.36 | 1.26 | 13 | 0.15 | 202 | 4.95 |
|  | Month 2 | 7.5 | 2.05 | 0.97 | 0.21 | 22 | <0.6 | ND | 0.59 | 1.36 | 19 | 0.21 | 309 | 3.02 |
|  | Month 3 | 10.2 | 2.71 | 1.52 | 0.35 | 78 | NA | NA | 0.56 | 2.09 | 15 | 0.17 | 298 | 0.65 |
|  | Month 4 | 11.8 | 2.75 | 1.29 | 0.61 | 70 | NA | NA | 0.51 | 3.19 | 12 | 0.14 | 289 | 0.56 |
|  | Month 6 | 11.9 | 2.07 | 0.88 | 0.67 | 96 | NA | NA | 0.51 | 2.83 | 12 | 0.14 | 274 | 0.39 |
|  | Month 9 | 13.0 | 5.59 | 3.20 | 1.58 | 143 | NA | NA | 0.51 | NA | 17 | 0.19 | ND | NA |
|  | Month 12 | 12.8 | 5.33 | 3.20 | 1.48 | 135 | NA | NA | 0.56 | ND | 19 | 0.21 | NA | 0.00 |

| Subject study number | Time  point | Hb  (g/dl) | WCC  (x10^9^/L) | ANC  (x10^9^/L) | ALC  (x10^9^/L) | Platelet  (x10^9^/L) | CRP  (mg/L) | Ferritin  (pmol/L) | ALT (IU/L)  (x ULN) | Fibrinogen  (g/L) | Creatinine  (μmol/L) | Creatinine  (mg/dL) | LDH (IU/L) | EASIX score |
| --- | --- | --- | --- | --- | --- | --- | --- | --- | --- | --- | --- | --- | --- | --- |
| CUCART-  006 | Screening | 9.8 | 0.83 | 0.43 | 0.37 | 30 | 26 | NA | 1.63 | ND | 29 | 0.33 | 397 | 4.34 |
|  | Pre cell collection | 9.1 | 4.86 | 3.32 | 0.85 | 316 | NA | NA | 0.37 | 2.62 | 46 | 0.52 | 306 | 0.50 |
|  | Day -4 | 10.5 | 3.41 | 2.63 | 0.08 | 350 | 5.1 | 506 | 0.31 | 2.80 | 40 | 0.45 | 208 | 0.27 |
|  | Day 0 | 10.5 | 1.08 | 0.92 | 0.02 | 317 | 1.0 | ND | 0.24 | 2.27 | 50 | 0.57 | 208 | 0.37 |
|  | Day 1 | 9.9 | 3.11 | 2.91 | 0.02 | 275 | 3.5 | 892 | 0.24 | 2.24 | 54 | 0.61 | 189 | 0.42 |
|  | Day 2 | 9.2 | 1.44 | 1.20 | 0.03 | 201 | 24 | 759 | 0.20 | 2.83 | 49 | 0.55 | 187 | 0.52 |
|  | Day 3 | 9.3 | 1.23 | 0.90 | 0.04 | 145 | 8.5 | 663 | 0.31 | 1.88 | 44 | 0.50 | 181 | 0.62 |
|  | Day 4 | 9.2 | 1.12 | 0.59 | 0.09 | 162 | 4.1 | 724 | 0.39 | 1.68 | 54 | 0.61 | 201 | 0.76 |
|  | Day 5 | 10.1 | 1.34 | 0.77 | 0.14 | 192 | 2.6 | 863 | 0.41 | 1.75 | 57 | 0.64 | 232 | 0.78 |
|  | Day 6 | 9.7 | 1.68 | 0.60 | 0.35 | 181 | 0.9 | 598 | 0.41 | 1.43 | 54 | 0.61 | 202 | 0.68 |
|  | Day 7 | 9.5 | 2.01 | 0.41 | 0.81 | 178 | 0.6 | 566 | 0.35 | 1.17 | 41 | 0.46 | 202 | 0.53 |
|  | Day 14 | 11.1 | 3.43 | 0.45 | 1.72 | 118 | <0.6 | 458 | 0.65 | 1.03 | 54 | 0.61 | 250 | 1.29 |
|  | Day 28 | 11.9 | 3.08 | 0.9 | 0.89 | 65 | <0.6 | 339 | 0.61 | 1.20 | 47 | 0.53 | 271 | 2.22 |
|  | Month 2 | 12.5 | 2.60 | 1.14 | 0.32 | 137 | NA | NA | 2.04 | ND | 42 | 0.48 | 279 | 0.97 |
|  | Month 3 | 13.3 | 4.18 | 2.57 | 0.47 | 182 | NA | NA | 0.76 | 3.22 | 48 | 0.54 | 265 | 0.79 |
|  | Month 4 | 13.2 | 6.41 | 4.36 | 0.68 | 211 | NA | NA | 0.57 | 3.60 | 48 | 0.54 | 268 | 0.69 |
|  | Month 6 | 13.7 | 6.54 | 4.91 | 0.58 | 206 | NA | NA | 0.49 | 4.07 | 54 | 0.61 | 250 | 0.74 |
|  | Month 9 | 14 | 6.01 | 4.25 | 0.82 | 203 | NA | NA | 0.45 | 4.00 | 46 | 0.52 | 267 | 0.68 |
|  | Month 12 | 13.4 | 6.79 | 4.94 | 0.95 | 219 | NA | NA | 20 | 3.48 | 43 | 0.49 | 241 | 0.54 |

| Subject study number | Time  point | Hb  (g/dl) | WCC  (x10^9^/L) | ANC  (x10^9^/L) | ALC  (x10^9^/L) | Platelet  (x10^9^/L) | CRP  (mg/L) | Ferritin  (pmol/L) | ALT (IU/L)  (x ULN) | Fibrinogen  (g/L) | Creatinine  (μmol/L) | Creatinine  (mg/dL) | LDH (IU/L) | EASIX score |
| --- | --- | --- | --- | --- | --- | --- | --- | --- | --- | --- | --- | --- | --- | --- |
| CUCART-  013 | Screening | 10.7 | 4.15 | 2.8 | 0.75 | 286 | ND | ND | 0.48 | ND | 20 | 0.23 | 219 | 0.17 |
|  | Pre cell collection | 9.3 | 3.13 | 1.25 | 1.25 | 202 | ND | ND | 0.41 | ND | 25 | 0.28 | ND | NA |
|  | Day -4 | 9.8 | 2.34 | 1.86 | 0.09 | 430 | 5.6 | 1317 | 0.45 | 3.64 | 28 | 0.32 | 216 | 0.16 |
|  | Day 0 | 8.9 | 1.15 | 1.00 | 0.03 | 300 | 0.7 | 1191 | 0.32 | 2.37 | 23 | 0.26 | 181 | 0.16 |
|  | Day 1 | 8.9 | 1.39 | 1.24 | 0.02 | 252 | 1.3 | 971 | 0.32 | 2.39 | 23 | 0.26 | 179 | 0.18 |
|  | Day 2 | 9.2 | 1.49 | 1.13 | 0.11 | 264 | 8.0 | 903 | 0.32 | 3.38 | 32 | 0.36 | 198 | 0.27 |
|  | Day 3 | 8.9 | 0.44 | 0.17 | 0.13 | 230 | 32 | 1081 | 0.30 | 3.41 | 30 | 0.34 | 191 | 0.28 |
|  | Day 4 | 9.3 | 0.41 | 0.08 | 0.13 | 225 | 15 | 1982 | 0.34 | 2.80 | 29 | 0.33 | 187 | 0.27 |
|  | Day 5 | 9.7 | 0.42 | 0.07 | 0.11 | 222 | 5.8 | 2094 | 0.30 | 2.57 | 25 | 0.28 | 208 | 0.26 |
|  | Day 6 | 10.0 | 0.66 | 0.13 | 0.30 | 219 | 3.5 | 1953 | 0.30 | 2.19 | 26 | 0.29 | 242 | 0.33 |
|  | Day 7 | 10.3 | 0.9 | 0.13 | 0.35 | 158 | 2.2 | 1959 | 0.36 | 2.10 | 20 | 0.23 | 267 | 0.38 |
|  | Day 13 | 10.7 | 1.31 | 0.39 | 0.62 | 169 | <0.6 | 1577 | 1.27 | 1.60 | 22 | 0.25 | 244 | 0.36 |
|  | Day 29 | 11.2 | 1.83 | 0.55 | 0.77 | 149 | <0.6 | 1045 | 0.93 | 1.41 | 22 | 0.25 | 256 | 0.43 |
|  | Month 2 | 11.2 | 1.97 | 0.88 | 0.67 | 165 | NA | NA | 0.55 | 1.52 | 19 | 0.21 | 218 | 0.28 |
|  | Month 3 | 12.0 | 2.19 | 0.65 | 1.11 | 203 | NA | NA | 0.48 | 2.71 | 24 | 0.27 | 255 | 0.34 |
|  | Month 4 | 11.8 | 2.04 | 0.21 | 1.46 | 203 | NA | NA | 0.36 | 2.61 | 24 | 0.27 | 224 | 0.30 |
|  | Month 6 | T/F | T/F | T/F | T/F | T/F | T/F | T/F | T/F | T/F | T/F | T/F | T/F | T/F |
|  | Month 9 | T/F | T/F | T/F | T/F | T/F | T/F | T/F | T/F | T/F | T/F | T/F | T/F | T/F |
|  | Month 12 | T/F | T/F | T/F | T/F | T/F | T/F | T/F | T/F | T/F | T/F | T/F | T/F | T/F |

| Subject study number | Time  point | Hb  (g/dl) | WCC  (x10^9^/L) | ANC  (x10^9^/L) | ALC  (x10^9^/L) | Platelet  (x10^9^/L) | CRP  (mg/L) | Ferritin  (pmol/L) | ALT (IU/L)  (x ULN) | Fibrinogen  (g/L) | Creatinine  (μmol/L) | Creatinine  (mg/dL) | LDH (IU/L) | EASIX score |
| --- | --- | --- | --- | --- | --- | --- | --- | --- | --- | --- | --- | --- | --- | --- |
| CUCART  -015 | Pre cell collection | 8.7 | 1.27 | 0.5 | 0.63 | 117 | ND | ND | 0.74 | ND | 23 | 0.26 | 144 | 0.32 |
|  | Day -4 | 9.3 | 4.09 | 3.15 | 0.53 | 71 | 3.1 | 5507 | 0.44 | 2.65 | 19 | 0.21 | 288 | 0.87 |
|  | Day 0 | 7.7 | 0.12 | 0.10 | 0.02 | 35 | 2.5 | 4532 | 0.51 | 2.48 | 17 | 0.19 | 156 | 0.86 |
|  | Day 1 | 6.2 | <0.08 | ND | ND | 21 | 1.6 | 3364 | 0.36 | 1.87 | 21 | 0.24 | 139 | 1.57 |
|  | Day 2 | 10.0 | <0.08 | ND | ND | 28 | 1.3 | 3422 | 0.38 | 1.89 | 25 | 0.28 | 150 | 1.52 |
|  | Day 3 | 7.6 | <0.08 | ND | ND | 21 | 0.7 | 5647 | 0.36 | 1.46 | 22 | 0.25 | 172 | 2.04 |
|  | Day 4 | 6.6 | 0.1 | ND | ND | 32 | <0.6 | 5930 | 0.44 | 1.68 | 27 | 0.31 | 194 | 1.85 |
|  | Day 5 | 9.3 | <0.08 | ND | ND | 18 | <0.6 | 20052 | 1.54 | 1.22 | 21 | 0.24 | 516 | 6.81 |
|  | Day 6 | 9.1 | 0.08 | ND | ND | 29 | <0.6 | 51755 | 1.62 | 1.35 | 20 | 0.23 | 642 | 5.01 |
|  | Day 7 | 7.6 | <0.08 | ND | ND | 28 | <0.6 | 114873 | 1.90 | 0.74 | 21 | 0.24 | 904 | 7.67 |
|  | Day 10 | 10.2 | 5.86 | 0.00 | 0.18 | 39 | <0.6 | >224700 | 3.46 | 0.67 | 22 | 0.25 | 10626 | 67.81 |
|  | Day 14 | 9.4 | 0.54 | 0.04 | 0.47 | 20 | <0.6 | 64682 | 1.67 | 1.17 | 9 | 0.10 | 1641 | 8.35 |
|  | Day 22 | 9.1 | 0.48 | 0.06 | 0.39 | 27 | <0.6 | 8172 | 1.33 | 1.24 | 13 | 0.15 | 292 | 1.59 |
|  | Day 29 | 7.6 | 0.38 | 0.10 | 0.19 | 26 | <0.6 | 5739 | 0.56 | 2.10 | 15 | 0.17 | 171 | 1.12 |
|  | Month 2 | 11.6 | 2.06 | 1.28 | 0.31 | 144 | <0.6 | 7244 | 0.67 | 1.66 | 15 | 0.17 | 241 | 0.28 |
|  | Month 3 | T/F | T/F | T/F | T/F | T/F | T/F | T/F | T/F | T/F | T/F | T/F | T/F | T/F |
|  | Month 4 | T/F | T/F | T/F | T/F | T/F | T/F | T/F | T/F | T/F | T/F | T/F | T/F | T/F |
|  | Month 6 | T/F | T/F | T/F | T/F | T/F | T/F | T/F | T/F | T/F | T/F | T/F | T/F | T/F |
|  | Month 9 | T/F | T/F | T/F | T/F | T/F | T/F | T/F | T/F | T/F | T/F | T/F | T/F | T/F |
|  | Month 12 | T/F | T/F | T/F | T/F | T/F | T/F | T/F | T/F | T/F | T/F | T/F | T/F | T/F |

| Subject study number | Time  point | Hb  (g/dl) | WCC  (x10^9^/L) | ANC  (x10^9^/L) | ALC  (x10^9^/L) | Platelet  (x10^9^/L) | CRP  (mg/L) | Ferritin  (pmol/L) | ALT (IU/L)  (x ULN) | Fibrinogen  (g/L) | Creatinine  (μmol/L) | Creatinine  (mg/dL) | LDH (IU/L) | EASIX score |
| --- | --- | --- | --- | --- | --- | --- | --- | --- | --- | --- | --- | --- | --- | --- |
| CUCART-  017 | Pre cell collection | 8.4 | 1.67 | 0.62 | 0.70 | 267 | ND | ND | 0.73 | ND | 33 | 0.37 | 207 | 0.29 |
|  | Day -4 | 9.9 | 3.53 | 3.32 | 0.04 | 330 | <0.6 | ND | 0.34 | 1.97 | 28 | 0.32 | 218 | 0.21 |
|  | Day 0 | 9.3 | 0.76 | 0.71 | 0.02 | 273 | <0.6 | 1726 | 0.41 | NA | 28 | 0.32 | 201 | 0.23 |
|  | Day 1 | 8.3 | 1.06 | 0.94 | 0.03 | 239 | <0.6 | 1425 | 0.68 | 1.82 | 29 | 0.33 | 189 | 0.26 |
|  | Day 2 | 8.3 | 1.04 | 0.84 | 0.05 | 221 | <0.6 | 1308 | 0.66 | 1.88 | 28 | 0.32 | 186 | 0.27 |
|  | Day 3 | 7.7 | 0.32 | 0.13 | 0.06 | 220 | 0.7 | 1157 | 0.61 | 1.82 | 30 | 0.34 | 172 | 0.27 |
|  | Day 4 | 8.0 | 0.28 | 0.07 | 0.05 | 219 | <0.6 | 1189 | 0.61 | 1.73 | 33 | 0.37 | 196 | 0.33 |
|  | Day 5 | 7.9 | 0.42 | 0.11 | 0.06 | 186 | <0.6 | 1225 | 0.59 | 2.05 | 33 | 0.37 | 190 | 0.38 |
|  | Day 6 | 8.6 | 1.00 | 0.39 | 0.11 | 225 | 2.9 | 1553 | 0.52 | 3.11 | 36 | 0.41 | 213 | 0.39 |
|  | Day 7 | 8.2 | 0.56 | 0.08 | 0.15 | 191 | 3.0 | 1573 | 0.55 | 2.9 | 36 | 0.41 | 219 | 0.47 |
|  | Day 14 | 9.3 | 1.41 | 0.36 | 0.60 | 117 | <0.6 | 1800 | 0.98 | 1.55 | 30 | 0.34 | 231 | 0.67 |
|  | Day 28 | 7.5 | 0.71 | 0.36 | 0.23 | 16 | <0.6 | 1368 | 0.25 | 1.15 | 29 | 0.33 | 218 | 4.50 |
|  | Month 2 | T/F | T/F | T/F | T/F | T/F | T/F | T/F | T/F | T/F | T/F | T/F | T/F | T/F |
|  | Month 3 | T/F | T/F | T/F | T/F | T/F | T/F | T/F | T/F | T/F | T/F | T/F | T/F | T/F |
|  | Month 4 | T/F | T/F | T/F | T/F | T/F | T/F | T/F | T/F | T/F | T/F | T/F | T/F | T/F |
|  | Month 6 | T/F | T/F | T/F | T/F | T/F | T/F | T/F | T/F | T/F | T/F | T/F | T/F | T/F |
|  | Month 9 | T/F | T/F | T/F | T/F | T/F | T/F | T/F | T/F | T/F | T/F | T/F | T/F | T/F |
|  | Month 12 | T/F | T/F | T/F | T/F | T/F | T/F | T/F | T/F | T/F | T/F | T/F | T/F | T/F |

ALT: alanine aminotransferase; CRP: C-reactive protein; Hb: haemoglobin; WCC: white cell count; ALC: absolute lymphocyte count; ANC: absolute neutrophil count; EASIX: endothelial activation and stress index; LDH: lactate dehydrogenase; NA: not available; ND: not detectable; T/F: to follow; ULN: upper limit normal
